# Molecular detection and phylogenetic analysis of pigeon circovirus from racing pigeons in Northern China

**DOI:** 10.1186/s12864-022-08425-8

**Published:** 2022-04-11

**Authors:** Haoran Wang, Hui Gao, Zhiwen Jiang, Leibo Shi, Pengwei Zhao, Yanming Zhang, Chengbao Wang

**Affiliations:** College of Veterinary Medicine, Northwest A&F University, Yangling, 712100 China

**Keywords:** Genetic diversity, Molecular epidemiology, Phylogenetic analysis, Pigeon circovirus, Sequence analysis

## Abstract

**Background:**

Pigeon circovirus (PiCV) infections in pigeons (*Columba livia*) have been reported worldwide. Currently, pigeon racing is becoming increasingly popular and considered to be a national sport in China, and even, the greatest competitions of racing pigeons are taking place in China. However, there are still no epidemiologic data regarding PiCV infections among racing pigeons in China. The purpose of our study was to provide information of prevalence, genetic variation and evolution of PiCV from racing pigeons in China.

**Results:**

To trace the prevalence, genetic variation and evolution of PiCV in sick and healthy racing pigeons, 622 samples were collected from 11 provinces or municipalities in China from 2016 to 2019. The results showed that the positive rate of PiCV was 19.3% (120/622) at the sample level and 59.0% (23/39) at the club level, thus suggesting that the virus was prevalent in Chinese racing pigeons. A sequence analysis revealed that the *cap* genes of the PiCV strains identified in our study displayed a high genetic diversity and shared nucleotide homologies of 71.9%–100% and amino acid homologies of 71.7%–100%. 28 and 36 unique amino acid substitutions were observed in the Cap and Rep proteins derived from our PiCV strains, respectively. A cladogram representation of PiCV strains phylogeny based on 90 *cap* gene sequences showed that the strains in this study could be further divided into seven clades (A, B, C, E, G, H, and I) and some of them were closely related to worldwide strains from different types of pigeons. A large number of recombination events (31 events) were also detected in the PiCV genomes from Chinese racing pigeons.

**Conclusions:**

These findings indicate that PiCV strains circulating in China exhibit a high genetic diversity and also contribute to information of prevalence, genetic variation and evolution of PiCV from racing pigeons in China.

**Supplementary Information:**

The online version contains supplementary material available at 10.1186/s12864-022-08425-8.

## Background

Members of the *Circoviridae*, within the order *Circovirus*, contain a small, circular, non-enveloped, single stranded DNA genome ranging from 1.7 to 2.5 kb in size [1]. The Pigeon circovirus (PiCV) or the Columbid circovirus (CoCV), together with Porcine circoviruses (PCV) types 1, 2, 3 and 4, Beak and feather disease virus (BFDV), Duck circovirus (DuCV), Goose circovirus (GoCV), Canary circovirus (CaCV), Raven circovirus (RaCV), Starling circovirus (StCV), Swan circovirus (SwCV), Finch circovirus (FiCV), and Gull circovirus (GuCV), etc. belong to the genus *Circovirus* of the family *Circoviridae* (http://www.ictvonline.org/). PiCV is a small, circular, non-enveloped virus that contains a single-stranded DNA genome with approximately 2 kb in size. The genome of PiCV has two main open reading frames (ORFs). The ORF-V1 located on the virion sense strand encodes a replication-associated protein (Rep), and the ORF–C1 located on the complementary sense strand encodes a capsid protein (Cap) [2, 3]. The gene forming ORF-C1 of PiCV has been demonstrated to be highly genetically diverse as compared with the gene forming ORF-V1 [1, 4]. The other ORFs including ORF-C2, ORF-C3, and ORF-C4 located on the complementary sense strand encode three viral proteins of unknown functions [2, 5]. The circovirus infection in pigeons was first identified in 1993 in the USA [6] and had been considered to be strongly associated with young pigeon disease syndrome (YPDS), including weakened racing performance, weight loss, lethargy, anorexia, respiratory distress, and diarrhea [7].

A variety of methods have been employed to detect PiCV infection in clinical specimens. The original diagnose methods electron microscopy, histology, dot blot hybridization, and in situ hybridization are time consuming [8–11]. The molecular biology techniques, such as the standard PCR [10, 12], Real-time PCR [13], NGS techniques [14], and the loop-mediated isothermal amplification method [11], enabled more rapid and accurate detection of PiCV infections. As a result, cases of PiCV infections in pigeons were subsequently reported in various countries, including Northern Ireland [3], Germany [2, 7], Italy [15], France [16], Czech Republic [17], Belgium [18, 19], Poland [20, 21], Slovenia [22], Hungary [23], United Arab Emirates [24], Iran [25], China [4, 14, 26], Japan [27], USA [6, 12], Brazil [28] and Australia [29]. Previous studies also demonstrated that PiCV had been detected in different types of pigeons, including racing, fancy, feral and meat pigeons [14, 20, 30]. However, all of the above-mentioned birds belong to *Columba livia* species.

In China, the first PiCV infection was detected from meat pigeons in Zhejiang province in 2009, and the full genome was sequenced [31]. In recent years, several studies have proved that PiCV was prevalent among meat pigeons in eastern and southern China [4, 14]. Recently, the competitions of racing pigeons are becoming increasingly popular in China, and even, pigeon racing is considered to be a national sport. The Chinese Association of Racing Pigeon Breeders has over 5 million members. Chinese breeders compete in numerous races for different distances in their sections throughout the racing season, and more than 25 million racing pigeons from approximately 750 racing clubs were selected to take part in the competitions every year. However, there are no epidemiologic data on PiCV infections among racing pigeons. In order to investigate the prevalence, evolution, and genome characterization of PiCV in racing pigeons in China, an extensive epidemiological investigation and bioinformatic analysis of PiCV from racing pigeons were undertaken in this study. The purpose of this study is to provide novel epidemiologic data and genome characterization for PiCV strains identified from racing pigeons in China.

## Results

### PCR detection and prevalence analysis of PiCV infection in Chinese racing pigeons

In this study, PiCV was detected in samples from both sick and healthy racing pigeons. The results indicated that the positive rates of PiCV were 19.3% (120/622) at the sample level. Among the samples, 14 out of 51 sick pigeons (positive rate, 27.5%) and 106 out of 571 healthy pigeons (positive rate, 18.6%) were identified to be PiCV positive, respectively. In this study, 23 out of 39 racing pigeon clubs were positive for PiCV (positive rate, 59.0%) (Table 1). Detailed information for 120 PiCV positive samples from racing pigeons in China is shown in Table 1.

**Table 1:** The results of PiCV detection in Chinese racing pigeons

Classification	Sick	Healthy	PiCV Positive number
	Pos/Tot (%)	Pos/Tot (%)	Pos/Tot (%)
Pigeon Club			
	5/7 (71.4%)	18/32 (56.3%)	23/39 (59.0%)
Geographic distributions			
Beijing	0/0 (0.0%)	1/19 (5.3%)	1/19 (5.3%)
Shaanxi	1/13 (7.7%)	101/466 (21.7%)	102/479 (21.3%)
Hebei	9/23 (39.1%)	1/25 (4.0%)	10/48 (20.8%)
Liaoning	0/0 (0.0%)	0/18 (0.0%)	0/18 (0.0%)
Inner Mongolia	0/0 (0.0%)	0/6 (0.0%)	0/6 (0.0%)
Shanxi	0/0 (0.0%)	0/11 (0.0%)	0/11 (0.0%)
Ningxia	0/0 (0.0%)	0/8 (0.0%)	0/8 (0.0%)
Gansu	2/10 (20%)	0/12 (0.0%)	2/22 (9.1%)
Qinghai	0/0 (0.0%)	3/6 (50.0%)	3/6 (50%)
Xinjiang	1/2 (50%)	0/0 (0.0%)	1/2 (50%)
Shandong	1/3 (33.3%)	0/0 (0.0%)	1/3 (33.3%)
Total	14/51 (27.5%)	106/571 (18.6%)	120/622 (19.3%)

### Genome characterization of PiCV genomes

A total of sixty-seven full genome sequences were obtained and deposited in GenBank with accession numbers: MW181925 to MW181991 (Table S2). The assembled circular whole genome sequences were detected as eleven sizes ranging from 2030 to 2045 nt with their *cap* gene ranging from 813 to 828 nt in length and *rep* gene of 948 or 954 nt as shown in Table S2. Among these variable genome length, the most common genome sizes were 2037 (*n* = 21) and 2042 (*n* = 13) nucleotides, respectively (Table S2). The sequence comparison among the 67 identified PiCV strains revealed nucleotide homologies of 84.2%–100% and these sequences exhibited nucleotide homologies of 83.0%–97.8% and 82.0%–98.3% as compared with Chinese PiCV reference strains and the ones from the other countries (Table S3).

### Analysis of nucleotide sequence of the *cap* genes and amino acid sequence of the Cap proteins

To explore the genetic diversity of PiCV strains, the *cap* genes of 90 PiCV strains from the 120 PiCV positive samples were sequenced. Detailed information for the 90 *cap* genes was shown in Table S2. The 90 *cap* genes were used to compare with the reference sequences from China and other countries, respectively. The results showed that the 90 *cap* genes ranged from 813 to 828 nt in length. Fourteen, thirteen, one, fifty-three, seven and two of the 90 *cap* nucleotide sequences were 813 nt, 816 nt, 819 nt, 822 nt, 825 nt, and 828 nt in length, encoding a Cap protein of 270, 271, 272, 273, 274, and 275 residues, respectively (Table S2). In addition, we found that ATT and GTG also existed in the position of the start codon site. The sequence comparison of the 90 identified *cap* genes revealed nucleotide homologies of 71.9%–100% and deduced amino acid homologies of 71.7%–100%, and the *cap* genes exhibited low sequence similarities with Chinese PiCV reference strains (73.0%–99.6% nucleotide identity, 72.3%–100% amino acid identity) and the PiCV reference strains from other countries (68.8%–98.4% nucleotide identity, 63.6%–100% amino acid identity) (Table S3).

**Fig. 1:**
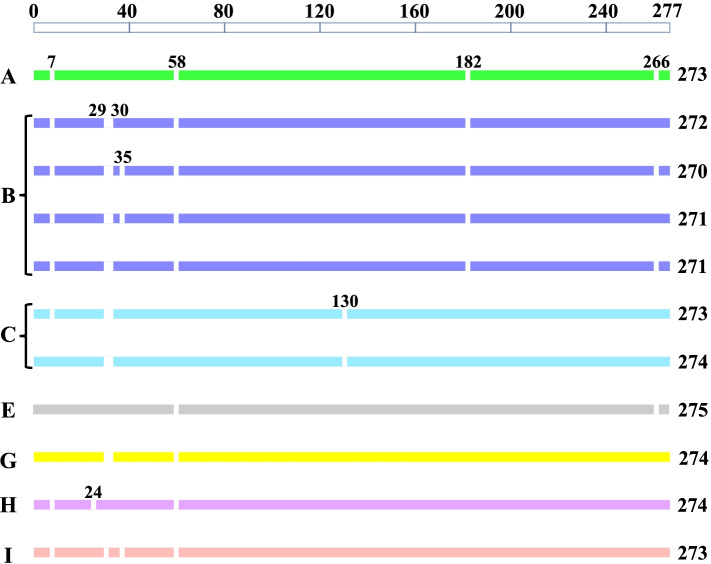
Size and amino acid sequence differences of 90 Cap proteins obtained in this study. Bars indicating the Cap proteins are filled according to the phylogenetic clade they occupy. The upper bar represents the consensus of the known PiCV Cap proteins; Amino acids are numbered from 1 to 277. Gaps indicate the location of deleted (compared to the consensus sequence) amino acids. The number on the right is the length of Cap proteins

**Fig. 2:**
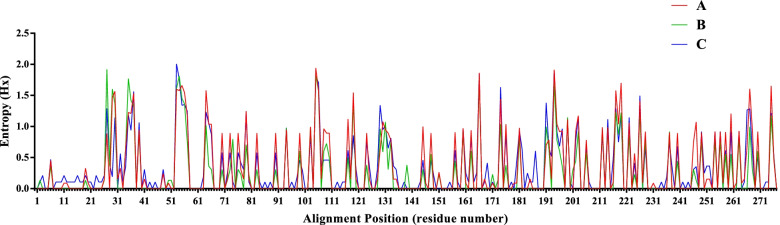
Comparison of the amino acid sequence conservation at a particular position between the 90 identified PiCV strains and the PiCV reference strains available in GenBank. **A**: The 90 identified PiCV strains from the racing pigeons in China. **B**: The 54 Chinese PiCV reference strains available in GenBank. **C**: The 72 PiCV reference strains available in GenBank from other countries. Note. The GenBank accession no. of the 54 Chinese PiCV reference strains, 72 PiCV reference strains from other countries, and the 90 identified PiCV strains was shown in Table S1 and S2

**Fig. 3:**
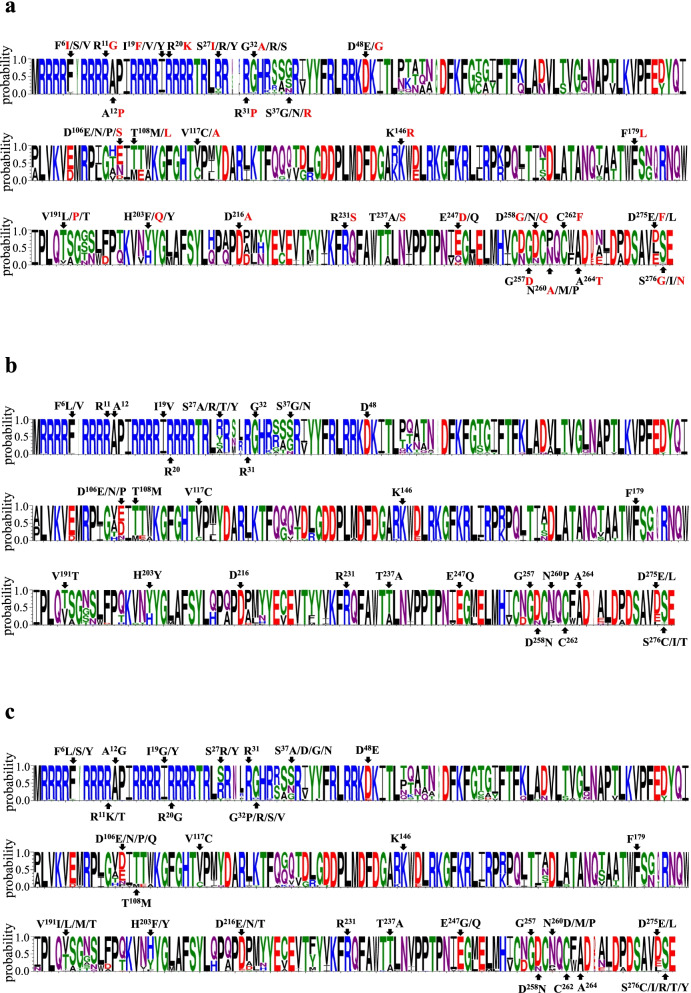
Divergence analysis of Cap proteins of the 90 identified PiCV strains. **a** The 90 identified PiCV strains from the racing pigeons in China. **b** The 54 Chinese PiCV reference strains available in GenBank. **c** The 72 PiCV reference strains available in GenBank from other countries. The unique amino acid mutations found in this study are indicated in red. Note. All PiCV reference strains were the same to the Fig. 2

To investigate variations in the deduced amino acid sequences, the amino acid sequences of 90 identified Cap proteins and the reference strains were aligned. The results showed that there were nine major locations of deletion (compared to the consensus sequence) among the Cap proteins including locations 7, 24, 29, 30, 35, 58, 130, 182, and 266 (Fig. 1). A comparison of entropy (Hx) in amino acid sequences of Cap proteins showed that the Hx of amino acid sequences at most positions from the 90 identified Cap proteins were higher than that of the PiCV reference strains (Fig. 2). In addition, some unique amino acid substitutions at 28 different positions were observed among the 90 Cap proteins as shown in Fig. 3.

### Analysis of nucleotide sequence of the *rep* genes and amino acid sequence of the Rep proteins

The *rep* genes for 68 out of 120 PiCV positive samples were successfully sequenced. Detailed information was shown in Table S2. The 68 identified *rep* genes were used to compare with PiCV reference sequences from China and other countries. The results showed that all of the 68 *rep* genes used the ATG start codon and were identified as two sizes: 948 and 954 nt. Forty of the 68 *rep* nucleotide sequences were 948 nt in length, encoding a Rep protein with 315 residues. Twenty-eight of the 68 *rep* nucleotide sequences were 954 nt in size, encoding a Rep protein with 317 residues (Table S2). The difference in size was due to a 2 amino acid deletion at positions 2 and 3. The sequence alignment revealed nucleotide homologies of 90.3%–100% and deduced amino acid homologies of 92.7%–100% among the 68 *rep* genes. These sequences exhibited higher similarities with PiCV reference strains from China (89.0%–99.2% nucleotide identity, 89.2%–99.6% amino acid identity) and other countries (89.5%–98.3% nucleotide identity, 90.5%–99.3% amino acid identity) as compared to the *cap* genes (Table S3). To investigate variations in the deduced amino acid sequences of *rep* gene products, the amino acid sequences of 68 identified *rep* genes and the reference strains were aligned. The results showed that some unique amino acid substitutions at 36 different positions were observed among the 68 identified PiCV strains (Data not shown).

### Phylogenetic analysis of the *cap* genes

**Fig. 4:**
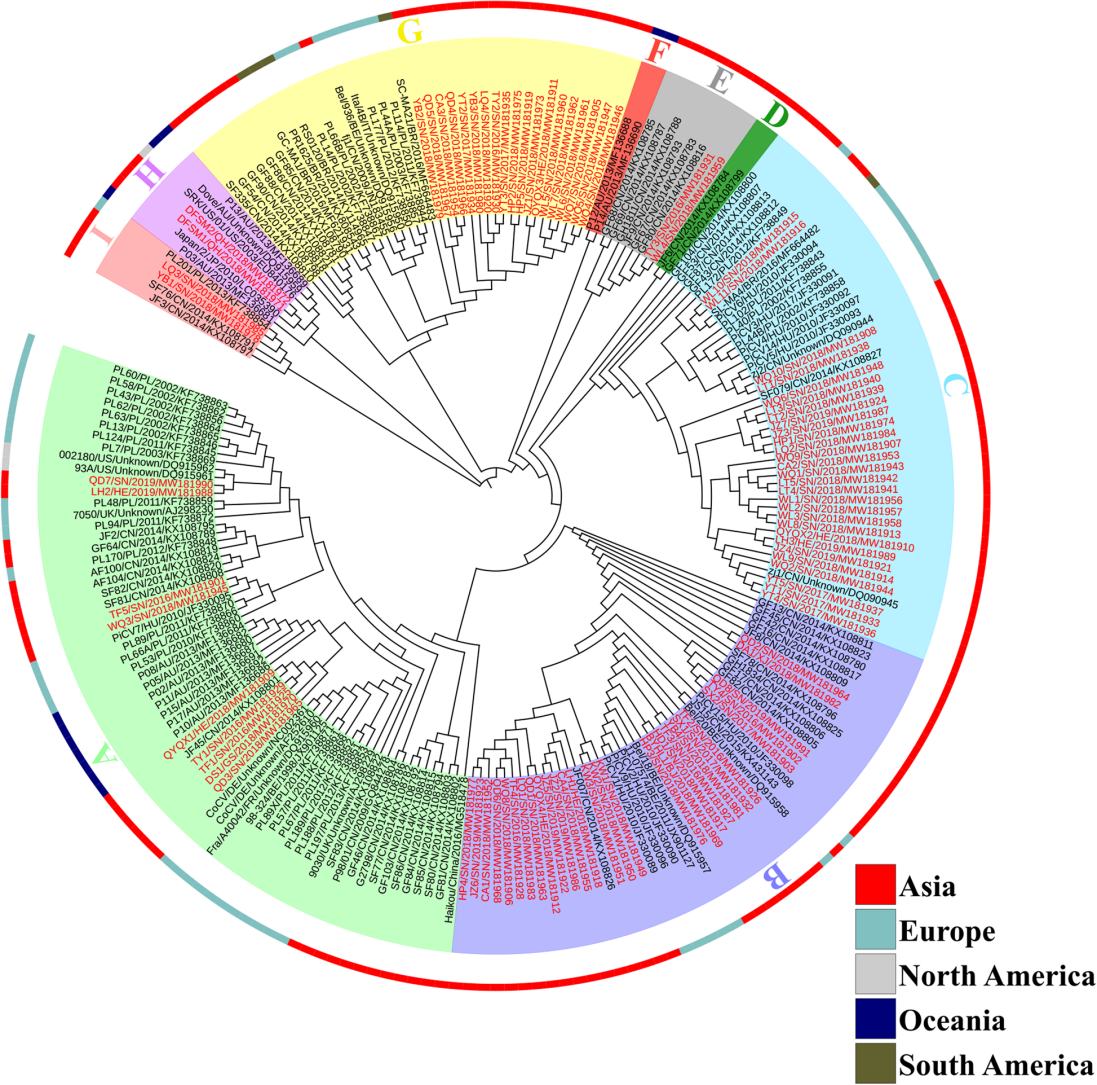
A cladogram representation of PiCV strains phylogeny based on 90 cap gene sequences identified in this study and 126 cap gene sequences available in GenBank. Labels at branch tips refer to the strain name and GenBank accession number. Red taxa highlight the 90 PiCV genome sequences isolated from the racing pigeons geographically located in China

A phylogenetic analysis using 126 *cap* gene reported in GenBank, and *cap* genes of 90 PiCV strains obtained in our study was performed to investigate genetic relationship (Table S1 and S2). The results indicated that a total of 216 analyzed PiCV strains could be arbitrarily divided into 9 clades, which were defined by the contractual letters A-I (Fig. 4). The 90 *cap* gene sequences reported in the present study were clustered into 7 clades: A (14.1%), B (60.9%), C (62.2%), E (25.0%), G (51.4%), H (28.6%), and I (40.0%). As shown in Fig. 4, the PiCV strains from different geographical locations were clustered into the same clades, such as QYQX1/HE/2018/MW181909, TY1/SN/2016/MW181929, DS1/GS/2018/MW181970, and QD3/SN/2018/MW181965. On the other hand, the PiCV strains from the same geographical locations were divided into different clades, such as LH1/HE/2018/MW181918 (clade B), LH2/HE/2019/MW181988 (clade A), and LH3/HE/2019/MW181989 (clade C). Furthermore, the PiCV strains collected from the same province belonged to different clades. For example, the 78 PiCV strains isolated from the pigeons in the racing clubs in Shannxi province, belonged to six separate clades except for clade D, F, and H.

### Recombination analysis in PiCV genomes

**Fig. 5:**
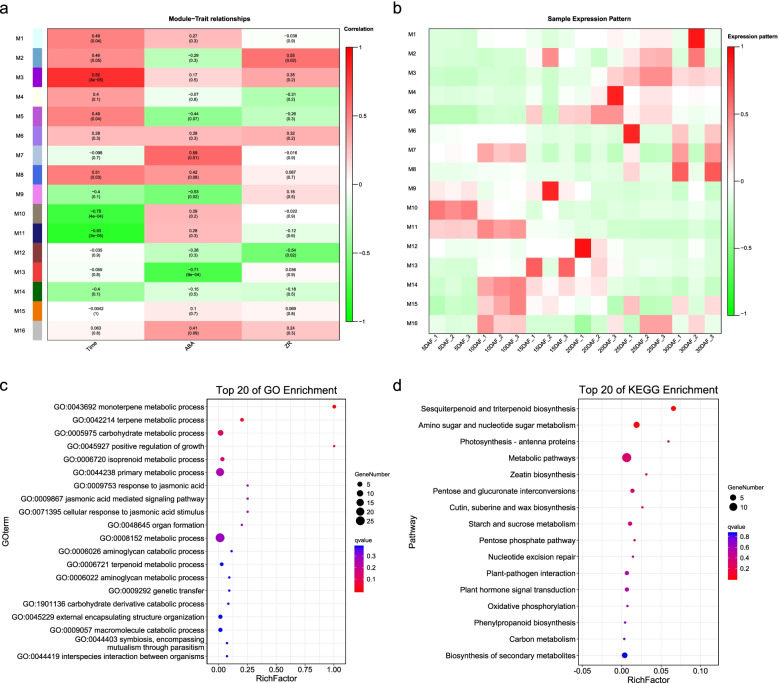
Recombination breakpoint distribution plots for 67 PiCV full genome sequences identified in this study and 113 PiCV full genome sequences available in GenBank. The red and blue areas of the plots indicate recombination breakpoint hot-spots and cold-spots, respectively. The dark and light grey areas represent the 95% and 99% confidence intervals of the expected degrees of breakpoint clustering under random recombination, respectively

For 67 identified PiCV strains, 31 recombination events were detected using RDP4 [32] (Table S4). These recombination events indicated that the strains originally from different types of pigeons or from different geographical locations may have some evidence of recombination. For example, a segment (~ 1001 bp) of three PiCV strains (LT3/SN/2018/MW181940, LT2/SN/2018/MW181939, and WQ6/SN/2018/MW181948) obtained in this study was likely descended from a large number of ancestral PiCV genomes that originated from Chinese meat pigeons, German racing pigeons, British racing pigeons, Polish fancy pigeons, and French meat pigeons (event 2). The possible breakpoints for recombination were determined during the recombination analysis. The results also indicated that the recombination breakpoint hot plots located within both the intergenic region between the *rep* and *cap* stop codons and near the virion strand origin of replication. Furthermore, the recombination breakpoint cold spots were detected within the central region of *cap* gene, which indicated few evidence for recombination within *cap* gene (Fig. 5). Additionally, four PiCV genomes (TF1/SN/2016/MW181925, DA1/XJ/2018/MW181982, QD7/SN/2019/MW181990, and LH2/HE/2019/MW181988) had no evidence for recombination.

## Discussion

In recent studies, Rotavirus A G18P [17] had been confirmed as a primary cause of YPDS-like diseases in domestic pigeons [33]. Furthermore, PiCV is also supposed to be an etiological agent of YPDS mostly affecting young pigeons worldwide [7]. PiCV has been reported to be prevalent in at least sixteen countries. In China, PiCV was first detected in meat pigeons in 2009 [31]. In recent years, the epidemiological survey showed that the positive rates of PiCV infection in Chinese meat pigeons were 19.67% and 75.3% in the poultry farms of China in 2009 and 2015 [4, 14], implying that PiCV is widely distributed among meat pigeon populations in China. However, the epidemiology and distribution of PiCV in the racing pigeons is unknown. The main objective of this work was to evaluate the genetic diversity and epidemiology of PiCV strains circulated in the racing pigeons of China. Positive samples were detected from seven provinces and the prevalence rates among these provinces were variant. Overall, our data implied that PiCV was also widely distributed in diseased racing pigeons and healthy racing pigeons in China for the first time.

Previous studies showed that the length of PiCV genome was 2031–2043 nt [14, 28, 29]. However, our findings first showed that the 2030 nt (*n* = 1), 2044 nt (*n* = 2), and 2045 nt (*n* = 1) complete genome were present in the PiCV positive samples. Despite many genetic diversities were found in the genome of PiCV strains, no mutation was found in the conserved nonanucleotide motif (TAGTATTAC) located at the apex of a potential stem-loop which was putatively associated with the initiation of rolling circle replication (RCR) [2, 3]. Previous studies have shown many genetic diversities in the *cap* gene [14, 23, 34]. Our findings showed that the 90 identified *cap* genes exhibited a higher diversity as compared with the reference strains. Some unique amino acid substitutions at 28 different positions were observed among the Cap proteins. In addition, the *cap* nucleotide sequence with 828 nt in length encoding a novel Cap protein of 275 amino acids was first identified in two Chinese PiCV strains, TY3/SN/2016/MW181931 (clade E) and WL4/SN/2018/MW181959 (clade E). Higher diversity of Cap protein versus the Rep protein is due to the fact that the Cap protein, as the protein shell of the virus, is exposed to the host’s immune cells, resulting in a stimulation of a cascade of immune responses. Positive selection occurs during this process. Some mutations can change the structure of Cap protein. This may lead to a better binding of virus with the receptors of target cells, which increases the infection ability of virus in cells. Moreover, these mutations may also protect virus from being neutralized by antibodies [1, 35, 36]. In this study, we identified many mutations in both N- and C-terminus of the Cap protein. According to the investigations on Cap protein of BFDV, the arginine-rich N-terminus has two functions (nuclear localization and nucleic acid binding) that enable as penetration of the viral genome into the host cell nucleus through nuclear pore complex [37]. Like BFDV, the N-terminus of PiCV Cap protein is also rich in arginine and has been predicted to be a nuclear localization signal and a nucleic acid binding domain by software [38], which means that this region is highly possible to be functionally similar to the corresponding region of the Cap protein of BFDV. Moreover, the results of this study showed that most of the amino acid deletion sites in Cap protein were concentrated in the N-terminus and many novel mutations also have been identified in this region. However, due to the lack of relevant experimental evidence, we cannot infer whether these mutations and deletions could affect the function of Cap protein. The Cap protein has been used as a coating antigen in ELISA due to its antigenic activity and can be recognized by PiCV specific antibodies [39, 40]. If the N-terminal region of the PiCV Cap protein, which is located within the capsid, resembles the corresponding region in BFDV, the N-terminal amino acid mutation and deletion may not affect the ability to bind to the antibody as a coating antigen [39]. In addition, there are also many mutations in the C-terminus where most sites are composed of hydrophilic amino acids. It means that this region may locate on the outside of the protein and may have ability for binding with antibody. Therefore, mutations of this region may lead to changes in antigenicity. Cap protein, which contains neutralizing antibody epitopes, can be considered as a potential antigen candidate in sub-unit vaccine development [41, 42]. Moreover, the sub-unit vaccines based on PCV2 recombinant capsid proteins are successfully used in the prevention of PCV2-SD [43–46]. It means that the development of a sub-unit vaccine may protect pigeons from infection with PiCV [42]. Due to a high genetic diversity of Cap proteins may lead to differences in antigenicity between different strains, sub-unit vaccines may also be diversified in future. To sum up, since there is little research on the function of PiCV Cap protein, the specific meaning of these mutations cannot be accurately explained. Therefore, further experiments are needed to explain if these mutations and deletions affect the function of Cap protein. According to the obtained nucleotide sequences of *cap* gene, besides ATG and ATA [2, 5, 14], we found that ATT and GTG also existed in the position of the start codon site through sequence alignment. Moreover, a similar situation has been found in GuCV [47] and BFDV [48]. Since no available cell lines have been found to culture PiCV in vitro [49, 50] and lack of commercial antibodies against PiCV Cap protein, it is hard to verify that indeed a full-length natural Cap protein is translated from the described alternative start codons. In all 68 identified Rep proteins, three amino acid motifs named FTLNNP (position 41–46), HLQGF (position 78–82), and YCSK (position 116–119) that putatively associated with RCR [2] were completely conserved. In addition, a fourth motif, which was putatively associated with dNTPase activity, namely GKS (position 197–199) [2], was also completely conserved. These sequence characteristics help understand the genetic diversity of PiCV. However, since the PCR amplification strongly relies on nucleotide homology in the regions where primers anneal, the samples containing more divergent viruses may not be obtained the full genome sequence when amplifying the viral genome. Thus, the actual diversity of circoviruses in Chinese pigeons may be even greater. This can be addressed by future studies employing next generation sequencing methods. Besides the pathogenicity of different PiCV strains still need to be validated in future.

In the present study, the 90 PiCV strains were divided into seven clades (A, B, C, E, G, H, and I) based on a phylogenetic analysis of *cap* gene. Additionally, we found that PiCV strains isolated from the same club belonged to different clades and shared a low sequence identity. These data suggested that the infection and evolution of PiCV in Chinese racing pigeons might have different evolutionary origins. This may result from the fact that the import of racing pigeons from all around the world to China is very significant and a lack of oversight in the international pigeon trade (racing pigeons mainly) [20, 51]. In addition, another possible explanation is that the intensive trafficking of pigeons between clubs and racing events in China. Interestingly, two isolates, QYQX1/HE/2018/MW181909 (clade A) and QYQX2/HE/2018/MW181910 (clade C), with lower identity (75.5% nucleotide identity and 76.8% amino acid identity) in the *cap* gene were detected from the same pigeon, suggesting a horizontal transmission occurred among the racing pigeons in the same racing clubs [6, 52]. The complexity of epidemic PiCV strains in China may cause difficulties to protect pigeon from infection by vaccines in future.

Viral recombination had been proved to play a significant role in the evolution of many ssDNA viruses [53]. The extensive recombination events had been reported in PiCV genomes and other circoviruses, such as PCV [54, 55] and BFDV [56, 57]. In this study, a large number of recombination events (31 events) were detected in the 67 identified PiCV strains. The high intensity of recombination may suggest that frequent co-infections of different PiCV strains. Moreover, recently, Khalifeh A. et al. has demonstrated that recombination plays a role in PiCV evolution through animal experiment [58]. Thus, the recombination seemed to be a key mechanism for PiCV evolution [21, 23, 28, 29].

## Conclusions

In conclusion, our study demonstrated that PiCV infection in racing pigeons was widespread in northern China and revealed the characteristics of the PiCV genome. Furthermore, the identified PiCV strains displayed a high genetic diversity. Our data also demonstrated that PiCV in Chinese racing pigeons had an extensive recombination for the first time. The high genetic diversity of PiCV may be derived from two possible facts: 1) the racing pigeons in China are imported from all around the world; and 2) a lack of oversight in the international pigeon trade (mainly refer to racing pigeons). In addition, another possible explanation is that the intensive trafficking of pigeons between clubs and racing events in China. Furthermore, the high intensity of recombination may suggest that frequent co-infections of different PiCV strains. Overall, clubs and racing events could provide a suitable chance for viral recombinants, which may pose a threat for the domestic pigeons, and has a possibility of spilling over onto wild species.

## Materials and methods

### Sample collection

In total, 622 samples were collected from 11 provinces or municipalities in China from November 2016 to August 2019. Of these samples, 571 serum samples were collected from 571 healthy racing pigeons from 39 racing pigeon clubs in 11 provinces or municipalities of China, including Beijing, Hebei, Liaoning, Shanxi, Gansu, Qinghai, Shandong, Shaanxi, Xinjiang Uygur Autonomous Region, Ningxia Hui Autonomous Region and Inner Mongolia Autonomous Region. Animals obtained from the racing pigeon clubs were returned to the club owners after collecting blood samples. Moreover, the corpses of 51 sick pigeons from 7 racing pigeon clubs in 5 provinces or municipalities were submitted to our laboratory at College of Veterinary Medicine, Northwest A&F University to determine the pathogen of the disease. The 0.2 g sections of internal organs (the heart, liver, spleen, lung, and intestine) were obtained during the postmortem examination of all mentioned dead pigeons. All samples were stored at − 80 °C before DNA extraction.

### DNA extraction

The tissue samples were ground to powder with liquid nitrogen and diluted with three volumes of phosphate‐buffered saline. The samples (1 ml) were centrifuged at 12,000 × g for 10 min at 4 °C and the supernatants were transferred into a 1.5 ml tube. Nucleic acids from tissues and serum samples were extracted by using an EasyPure® Viral DNA/RNA Kit (TransGen Biotech, Beijing, China) according to the manufacturer’s instructions. The extracted genomic DNA was stored at -20 °C before use.

### PCR detection of PiCV

The PiCV was first detected using a PCR method targeting a 326-base fragment of *cap* gene as described by Freick et al. (2008). The primer sequences were: PiCV-s, 5’-TTGAAAGGTTTTCAGCCTGGC-3’ and PiCV-as, 5’-AGGAGACGAAGGACACGCCTC-3’ [59]. The full genomes of PiCV for all the positive samples were amplified by PCR using the primers: PiCV-1F, 5’-ACCCGCGACTTGGAGCCACGGAG-3’ and PiCV-1R, 5’-TTCGCTCCCGCATTCGCGGTCGCT-3’; PiCV-2F, 5’-GACACTAGTAAAGGGACCCAAGCCA-3’ and PiCV-2R, 5’-AAGCCTTGCAGATGCGGGGT-3’, respectively. PCR was performed by using Q5 Hot Start High-Fidelity 2 × Master Mix (NEB, MA, USA). The contents of the three reactions mixture in a 50 μl reaction volume were as follows: 0.5 μM forward primer, 0.5 μM reverse primer, 1 μg genomic DNA, 25 μl Q5 High-Fidelity 2 × Master Mix (NEB, MA, USA) and an appropriate volume of Nuclease-Free Water. The cycling parameters were 30 cycles of 98 °C for 10 s, 55 °C for 30 s and 72 °C for 30 s, followed by a final extension at 72 °C for 5 min using an automated BioRad T100 Thermal Cycler (Bio-Rad Laboratories, Inc., CA, USA). The PCR products (5 μl) were resolved on 1% (w/v) agarose gels, and followed by staining with ethidium bromide. Finally, the bands of nucleic acid were visualized with UV illumination inside a gel documentation apparatus (Bio-Rad Laboratories, Inc., CA, USA) and saved as digital photographs.

### Genome characterization and sequence analysis of the *cap*, *rep* genes and PiCV genome

The *cap* gene sequences, *rep* gene sequence and the full genome sequences of PiCV obtained in this study have been deposited in GenBank, respectively. The sequences obtained in the current study were compared with all available PiCV sequence data from different geographical locations within China (*n* = 60) and the rest of the world (*n* = 73) from the National Center for Biotechnology Information (NCBI) nucleotide database (Table S1). Multiple sequence alignments were carried out by ClustalW algorithm using MEGA 5.0 software [60] and the homology among nucleotide and amino acid sequences was determined by using BioEdit v. 7.0.5 software [61]. A divergence analysis of the Cap protein of PiCV was performed using WebLogo^Tm^ (http://weblogo.threeplusone.com/), an online software for sequence logo generator [62]. A comparison of Hx in amino acid sequences of Cap proteins of PiCV was carried out by using GraphPad Prism 7.0 software (GraphPad Software, Inc., USA).

### Phylogenetic analysis

The entire *cap* gene sequences of PiCV obtained in this study were used for a phylogenetic analysis. All available PiCV sequence data from different geographical locations within China (*n* = 54) and the rest of the world (*n* = 72) were retrieved from the NCBI nucleotide database as reference sequences (Table S1). Maximum likelihood phylogenetic trees were constructed with the GTR + G + I model by using the MEGA 5.0 software [60], with partial deletion to handle alignment gaps and 1,000 bootstrap iterations. The cladogram was generated and annotated with the Interactive Tree Of Life (iTOL) software (http://itol.embl.de/) [63].

### Recombination analysis

In order to analyze the potential recombinant evidence, an integrated software package for the recombination detection program RDP4 [32] was used to detect potential recombinant strains, parental strains, and possible recombination breakpoints. Seven methods (RDP [64], GENECONV [65], BootScan [66], MaxChi [67], Chimaera [68], SiScan [69], and 3Seq [70]) were implemented using the RDP4 program [32]. Recombination events were identified by at least three of the aforementioned methods, and the *p*-value < 0.05 were considered plausible recombinant events. The sequences in the analyzed dataset that most closely resembled the parental sequences of recombinants were defined as either “minor parents” or “major parents” based on the size of the genome fragments which had contributed to the detected recombinants (with the major parent contributing the larger fragment and the minor parent the smaller).

## Supplementary Information

**Additional file 1: Table S1.** Pigeon circovirus (PiCV) references sequences obtained from GenBank and used in this study. The information including strain name, year, country, host, genome length and accession number.

**Additional file 2: Table S2.** Information of Pigeon circovirus (PiCV) stains obtained in this study. The information including strain name, collection date, collection region, gene length, initiation codons of *cap* gene, health status and accession number.

**Additional file 3: Table S3.** Sequence analysis of genome sequences of Pigeon circovirus (PiCV) strains. The information including homology analysis of amino acid and nucleotide sequences.

**Additional file 4: Table S4.** Details of recombination events (GenBank accession numbers) detected in the Pigeon circovirus (PiCV) strains obtained in this study. The information including location of recombination breakpoints, recombinant, potential minor parent(s), potential major parent(s), detection methods and *p*-value.

## Data Availability

The nucleotide sequence data obtained in this study are available in GenBank, and their accession numbers are MW181901- MW181991. The information of PiCV references sequences is shown in Table S1.

## Abbreviations

PiCV: Pigeon circovirus
Rep: Replication-associated protein
Cap: Capsid protein
CoCV: Columbid circovirus
PCV: Porcine circoviruses
BFDV: Beak and feather disease virus
DuCV: Duck circovirus
GoCV: Goose circovirus
CaCV: Canary circovirus
RaCV: Raven circovirus
StCV: Starling circovirus
SwCV: Swan circovirus
FiCV: Finch circovirus
GuCV: Gull circovirus
ORFs: Open reading frames
YPDS: Young pigeon disease syndrome
RCR: Rolling circle replication
NCBI: National Center for Biotechnology Information
