# Supplementary material for: Molecular detection and phylogenetic analysis of pigeon circovirus from racing pigeons in Northern China
Source: BMC Genomics. 2022 Apr 11;23:290. doi: 10.1186/s12864-022-08425-8 (PMC8995411; doi:10.1186/s12864-022-08425-8)
Supplement: Supplementary file 1 — Additional file 1: Table S1. Pigeon circovirus (PiCV) references sequences obtained from GenBank and used in this study. The information including strain name, year, country, host, genome length and accession number. [file 12864_2022_8425_MOESM1_ESM.docx]

**Supplementary Table 1** Pigeon circovirus (PiCV) references sequences obtained from GenBank and used in this study.

| Strain name | Year | Country | Host | Genome length (nt) | Accession number  (Genome sequence) | Accession number  (*cap* gene sequence) | Accession number  (*rep* gene sequence) |
| --- | --- | --- | --- | --- | --- | --- | --- |
| PiCV/Belgium/98-324/1998(*rep*) | 1998 | Belgium | pigeon | 954 |  |  | JX901125 |
| PiCV/Belgium/98-324/1998(*cap*) | 1998 | Belgium | pigeon | 822 |  | JX901125 |  |
| CoCV | unknown | Germany | racing pigeon | 2037 | AF252610 |  |  |
| CoCV | unknown | Germany | racing pigeon | 2037 | NC002361 |  |  |
| PL13 | 2002 | Poland | feral pigeon | 2041 | KF738846 |  |  |
| PL14 | 2002 | Poland | feral pigeon | 2034 | KF738847 |  |  |
| PL40 | 2002 | Poland | feral pigeon | 2042 | KF738855 |  |  |
| PL43 | 2002 | Poland | feral pigeon | 2038 | KF738856 |  |  |
| PL44B | 2002 | Poland | feral pigeon | 2043 | KF738858 |  |  |
| PL58 | 2002 | Poland | feral pigeon | 2038 | KF738862 |  |  |
| PL60 | 2002 | Poland | feral pigeon | 2038 | KF738863 |  |  |
| PL62 | 2002 | Poland | feral pigeon | 2038 | KF738864 |  |  |
| PL63 | 2002 | Poland | feral pigeon | 2041 | KF738865 |  |  |
| PL66B | 2002 | Poland | feral pigeon | 2034 | KF738867 |  |  |
| SRK/US/01 | 2003 | America | feral pigeon | 2041 | EU840176 |  |  |
| PL7 | 2003 | Poland | carrier pigeon | 2037 | KF738869 |  |  |
| PL44A | 2003 | Poland | carrier pigeon | 2038 | KF738857 |  |  |
| fj1 | 2009 | Fujian/China | carrier pigeon | 2037 | JN183455 |  |  |
| P98/01 | 2009 | Taiwan/China | pigeon | 822 |  | GQ844278 |  |
| PiCV1 | 2010 | Hungary | racing pigeon | 813 |  | JF330089 |  |
| PiCV2 | 2010 | Hungary | racing pigeon | 813 |  | JF330090 |  |
| PiCV3 | 2010 | Hungary | racing pigeon | 822 |  | JF330091 |  |
| PiCV4 | 2010 | Hungary | racing pigeon | 822 |  | JF330092 |  |
| PiCV5 | 2010 | Hungary | racing pigeon | 822 |  | JF330093 |  |
| PiCV6 | 2010 | Hungary | racing pigeon | 822 |  | JF330094 |  |
| PiCV7 | 2010 | Hungary | racing pigeon | 822 |  | JF330095 |  |
| PiCV9 | 2010 | Hungary | racing pigeon | 813 |  | JF330096 |  |
| PiCV14 | 2010 | Hungary | racing pigeon | 822 |  | JF330097 |  |
| PiCV15 | 2010 | Hungary | racing pigeon | 813 |  | JF330098 |  |
| PiCV/Japan/2/2010 | 2010 | Japan | racing pigeon | 2041 | LC035390 |  |  |
| PiCV/Belgium/11-07574/2011(*cap*) | 2011 | Belgium | pigeon | 813 |  | JX901127 |  |
| PL48 | 2011 | Poland | carrier pigeon | 2037 | KF738859 |  |  |
| PL53 | 2011 | Poland | carrier pigeon | 2035 | KF738860 |  |  |
| PL57 | 2011 | Poland | fancy pigeon | 2037 | KF738861 |  |  |
| PL66A | 2011 | Poland | fancy pigeon | 2037 | KF738866 |  |  |
| PL67 | 2011 | Poland | fancy pigeon | 2037 | KF738868 |  |  |
| PL89 | 2011 | Poland | fancy pigeon | 2037 | KF738870 |  |  |
| PL89X | 2011 | Poland | fancy pigeon | 2037 | KF738871 |  |  |
| PL94 | 2011 | Poland | carrier pigeon | 2037 | KF738872 |  |  |
| PL102 | 2011 | Poland | fancy pigeon | 2040 | KF738843 |  |  |
| PL114 | 2011 | Poland | fancy pigeon | 2038 | KF738844 |  |  |
| PL124 | 2011 | Poland | fancy pigeon | 2037 | KF738845 |  |  |
| PL170 | 2012 | Poland | farrier pigeon | 2036 | KF738848 |  |  |
| PL172 | 2012 | Poland | carrier pigeon | 2041 | KF738849 |  |  |
| PL177 | 2012 | Poland | carrier pigeon | 2037 | KF738850 |  |  |
| PL188 | 2012 | Poland | fancy pigeon | 2036 | KF738851 |  |  |
| PL189 | 2012 | Poland | fancy pigeon | 2036 | KF738852 |  |  |
| PiCV/P02/AUS | 2013 | Australia | feral pigeon | 2037 | MF136680 |  |  |
| PiCV/P03/AUS | 2013 | Australia | feral pigeon | 2039 | MF136681 |  |  |
| PiCV/P05/AUS | 2013 | Australia | feral pigeon | 2037 | MF136682 |  |  |
| PiCV/P08/AUS | 2013 | Australia | feral pigeon | 2037 | MF136684 |  |  |
| PiCV/P10/AUS | 2013 | Australia | feral pigeon | 2037 | MF136686 |  |  |
| PiCV/P11/AUS | 2013 | Australia | feral pigeon | 2037 | MF136687 |  |  |
| PiCV/P12/AUS | 2013 | Australia | feral pigeon | 2034 | MF136688 |  |  |
| PiCV/P13/AUS | 2013 | Australia | feral pigeon | 2039 | MF136689 |  |  |
| PiCV/P14/AUS | 2013 | Australia | feral pigeon | 2033 | MF136690 |  |  |
| PiCV/P15/AUS | 2013 | Australia | feral pigeon | 2037 | MF136691 |  |  |
| PiCV/P17/AUS | 2013 | Australia | feral pigeon | 2037 | MF136692 |  |  |
| AHBZ (*rep*) | 2013 | China | meat pigeon | 954 |  |  | KJ704801 |
| HBLF-E2 (*rep*) | 2013 | China | meat pigeon | 954 |  |  | KJ704802 |
| JSNJ (*rep*) | 2013 | China | meat pigeon | 954 |  |  | KJ704803 |
| NJPK (*rep*) | 2013 | China | meat pigeon | 954 |  |  | KJ704804 |
| SDDZ (*rep*) | 2013 | China | meat pigeon | 954 |  |  | KJ704805 |
| SHWH (*rep*) | 2013 | China | meat pigeon | 954 |  |  | KJ704806 |
| PL197 | 2013 | Poland | fancy pigeon | 2036 | KF738853 |  |  |
| PL201 | 2013 | Poland | fancy pigeon | 2043 | KF738854 |  |  |
| AF100 | 2014 | Anhui/China | meat pigeon | 2037 | KX108819 |  |  |
| AF104 | 2014 | Anhui/China | meat pigeon | 2037 | KX108824 |  |  |
| PR1625 | 2014 | Brazil | domestic pigeon | 2041 | KX808543 |  |  |
| RS0120 | 2014 | Brazil | domestic pigeon | 2041 | KY114965 |  |  |
| GF82 | 2014 | Guangdong/China | meat pigeon | 2031 | KX108805 |  |  |
| GF42 | 2014 | Guangdong/China | meat pigeon | 2037 | KX108780 |  |  |
| GF86 | 2014 | Guangdong/China | meat pigeon | 2034 | KX108781 |  |  |
| GF67 | 2014 | Guangdong/China | meat pigeon | 2040 | KX108783 |  |  |
| GF68 | 2014 | Guangdong/China | meat pigeon | 2037 | KX108785 |  |  |
| G2798 | 2014 | Guangdong/China | meat pigeon | 2037 | KX108786 |  |  |
| GF69 | 2014 | Guangdong/China | meat pigeon | 2037 | KX108787 |  |  |
| GH1811 | 2014 | Guangdong/China | meat pigeon | 2037 | KX108788 |  |  |
| GF85 | 2014 | Guangdong/China | meat pigeon | 2034 | KX108790 |  |  |
| GF103 | 2014 | Guangdong/China | meat pigeon | 2037 | KX108792 |  |  |
| GF54 | 2014 | Guangdong/China | meat pigeon | 2031 | KX108798 |  |  |
| GF71 | 2014 | Guangdong/China | meat pigeon | 2031 | KX108799 |  |  |
| GF104 | 2014 | Guangdong/China | meat pigeon | 2037 | KX108800 |  |  |
| GF81 | 2014 | Guangdong/China | meat pigeon | 2037 | KX108802 |  |  |
| GF46 | 2014 | Guangdong/China | meat pigeon | 2037 | KX108804 |  |  |
| GF17 | 2014 | Guangdong/China | meat pigeon | 2031 | KX108806 |  |  |
| GF53 | 2014 | Guangdong/China | meat pigeon | 2031 | KX108807 |  |  |
| GF13 | 2014 | Guangdong/China | meat pigeon | 2031 | KX108811 |  |  |
| GF43 | 2014 | Guangdong/China | meat pigeon | 2034 | KX108812 |  |  |
| GF87 | 2014 | Guangdong/China | meat pigeon | 2034 | KX108813 |  |  |
| GF84 | 2014 | Guangdong/China | meat pigeon | 2037 | KX108815 |  |  |
| GF80 | 2014 | Guangdong/China | meat pigeon | 2040 | KX108816 |  |  |
| GF16 | 2014 | Guangdong/China | meat pigeon | 2031 | KX108817 |  |  |
| GF90 | 2014 | Guangdong/China | meat pigeon | 2034 | KX108818 |  |  |
| GF88 | 2014 | Guangdong/China | meat pigeon | 2034 | KX108821 |  |  |
| GF45 | 2014 | Guangdong/China | meat pigeon | 2031 | KX108823 |  |  |
| GH1834 | 2014 | Guangdong/China | meat pigeon | 2031 | KX108825 |  |  |
| GF67 | 2014 | Jiangsu/China | meat pigeon | 2037 | KX108784 |  |  |
| JF9 | 2014 | Jiangsu/China | meat pigeon | 2040 | KX108793 |  |  |
| JF2 | 2014 | Jiangsu/China | meat pigeon | 2037 | KX108795 |  |  |
| JF3 | 2014 | Jiangsu/China | meat pigeon | 2040 | KX108797 |  |  |
| JF45 | 2014 | Jiangsu/China | meat pigeon | 2037 | KX108801 |  |  |
| JF8 | 2014 | Jiangsu/China | meat pigeon | 2031 | KX108809 |  |  |
| JF007 | 2014 | Jiangsu/China | meat pigeon | 2031 | KX108826 |  |  |
| GF64 | 2014 | Shanghai/China | meat pigeon | 2037 | KX108789 |  |  |
| SF76 | 2014 | Shanghai/China | meat pigeon | 2037 | KX108791 |  |  |
| SF85 | 2014 | Shanghai/China | meat pigeon | 2037 | KX108794 |  |  |
| SF78 | 2014 | Shanghai/China | meat pigeon | 2031 | KX108796 |  |  |
| SF80 | 2014 | Shanghai/China | meat pigeon | 2037 | KX108803 |  |  |
| SF81 | 2014 | Shanghai/China | meat pigeon | 2037 | KX108808 |  |  |
| SF335 | 2014 | Shanghai/China | meat pigeon | 2037 | KX108810 |  |  |
| SF86 | 2014 | Shanghai/China | meat pigeon | 2037 | KX108814 |  |  |
| SF82 | 2014 | Shanghai/China | meat pigeon | 2038 | KX108820 |  |  |
| SF83 | 2014 | Shanghai/China | meat pigeon | 2037 | KX108822 |  |  |
| SF079 | 2014 | Shanghai/China | meat pigeon | 2034 | KX108827 |  |  |
| SF77 | 2014 | Shanghai/China | meat pigeon | 2037 | KX108782 |  |  |
| JS15-1 | 2015 | China | racing pigeon | 2034 | KX431143 |  |  |
| SL-MA4/2016 | 2016 | Brazil | domestic pigeon | 2040 | MF664482 |  |  |
| SC-MA21/2016 | 2016 | Brazil | domestic pigeon | 2041 | MF664483 |  |  |
| Haikou | 2016 | Haikou/China | pigeon | 2038 | MG518478 |  |  |
| GC-MA1/2016 | 2016 | Brazil | domestic pigeon | 2038 | MF621931 |  |  |
| US 93A | Unknown | America | ornamental pigeon | 2037 | DQ915961 |  |  |
| US 002180 | Unknown | America | feral pigeon | 2036 | DQ915962 |  |  |
| Dove | Unknown | Australia | Senegal dove | 2039 | DQ915959 |  |  |
| Bel 18 | Unknown | Belgium | racing pigeon | 2032 | DQ915957 |  |  |
| Bel 20 | Unknown | Belgium | racing pigeon | 2032 | DQ915958 |  |  |
| Bel 936 | Unknown | Belgium | racing pigeon | 2038 | DQ915956 |  |  |
| Fra A40042 | Unknown | France | meat pigeon | 2037 | DQ915960 |  |  |
| Ita 4B | Unknown | Italy | meat pigeon | 2040 | DQ915950 |  |  |
| 7050 | Unknown | United Kingdom | racing pigeon | 2036 | AJ298230 |  |  |
| 9030 | Unknown | United Kingdom | racing pigeon | 2037 | AJ298229 |  |  |
| zj1 | Unknown | Zhejiang/China | meat pigeon | 2039 | DQ090945 |  |  |
| zj2 | Unknown | Zhejiang/China | meat pigeon | 2039 | DQ090944 |  |  |
