# Supplementary material for: Molecular detection and phylogenetic analysis of pigeon circovirus from racing pigeons in Northern China
Source: BMC Genomics. 2022 Apr 11;23:290. doi: 10.1186/s12864-022-08425-8 (PMC8995411; doi:10.1186/s12864-022-08425-8)
Supplement: Supplementary file 2 — Additional file 2: Table S2. Information of Pigeon circovirus (PiCV) stains obtained in this study. The information including strain name, collection date, collection region, gene length, initiation codons of cap gene, health status and accession number. [file 12864_2022_8425_MOESM2_ESM.docx]

**Supplementary Table** **2** Information of Pigeon circovirus (PiCV) stains obtained in this study. All sequence information can be found in the databases at the National Center for Biotechnical Information (NCBI) (<http://ncbi.nlm.nih.gov/>) by accession number.

| No. | Strain name | Club name | Collection date | Collection region | Genome length (nt) | *rep* gene length (nt) | *cap* gene length (nt) | Initiation codons of *cap* gene | Health status | Accession number |
| --- | --- | --- | --- | --- | --- | --- | --- | --- | --- | --- |
| 1 | TF5/SN/2016 | Teng Fei | 2016.11 | Shaanxi/China |  |  | 822 | GTG | Healthy | MW181901 |
| 2 | TY4/SN/2016 | Tian Ying | 2016.12 | Shaanxi/China |  |  | 816 | ATG | Healthy | MW181902 |
| 3 | SX2/SN/2017 | Sheng Xiang | 2017.05 | Shaanxi/China |  |  | 816 | ATG | Healthy | MW181903 |
| 4 | LT6/SN/2018 | Long Teng | 2018.03 | Shaanxi/China |  | 954 |  |  | Healthy | MW181904 |
| 5 | WQ7/SN/2018 | Wei Qi | 2018.03 | Shaanxi/China |  |  | 822 | ATG | Healthy | MW181905 |
| 6 | WQ8/SN/2018 | Wei Qi | 2018.03 | Shaanxi/China |  |  | 819 | ATG | Healthy | MW181906 |
| 7 | WQ9/SN/2018 | Wei Qi | 2018.03 | Shaanxi/China |  |  | 822 | ATG | Healthy | MW181907 |
| 8 | WQ10/SN/2018 | Wei Qi | 2018.03 | Shaanxi/China |  |  | 822 | ATG | Healthy | MW181908 |
| 9 | QYQX1/HE/2018 | Qian Yu Qian Xiang | 2018.04 | Hebei/China |  |  | 822 | ATA | Sick | MW181909 |
| 10 | QYQX2/HE/2018 | Qian Yu Qian Xiang | 2018.04 | Hebei/China |  |  | 822 | ATG | Sick | MW181910 |
| 11 | QYQX3/HE/2018 | Qian Yu Qian Xiang | 2018.04 | Hebei/China |  |  | 822 | ATG | Sick | MW181911 |
| 12 | QYQX4/HE/2018 | Qian Yu Qian Xiang | 2018.04 | Hebei/China |  |  | 813 | ATG | Sick | MW181912 |
| 13 | WL8/SN/2018 | Wei Li | 2018.05 | Shaanxi/China |  |  | 822 | ATG | Healthy | MW181913 |
| 14 | WL9/SN/2018 | Wei Li | 2018.05 | Shaanxi/China |  |  | 822 | ATG | Healthy | MW181914 |
| 15 | WL10/SN/2018 | Wei Li | 2018.05 | Shaanxi/China |  |  | 825 | ATG | Healthy | MW181915 |
| 16 | WL11/SN/2018 | Wei Li | 2018.05 | Shaanxi/China |  |  | 825 | ATG | Healthy | MW181916 |
| 17 | HD1/SN/2018 | Hua Du | 2018.07 | Shaanxi/China |  |  | 813 | ATG | Healthy | MW181917 |
| 18 | LH1/HE/2018 | Ling Hang | 2018.08 | Hebei/China |  |  | 816 | ATG | Healthy | MW181918 |
| 19 | HP5/SN/2018 | Hao Peng | 2018.09 | Shaanxi/China |  |  | 822 | ATG | Healthy | MW181919 |
| 20 | LQ4/SN/2018 | Li Quan | 2018.12 | Shaanxi/China |  |  | 822 | ATG | Healthy | MW181920 |
| 21 | JZ4/SN/2019 | Jin Zhou | 2019.02 | Shaanxi/China |  |  | 822 | ATG | Healthy | MW181921 |
| 22 | JZ5/SN/2019 | Jin Zhou | 2019.02 | Shaanxi/China |  |  | 816 | ATG | Healthy | MW181922 |
| 23 | JZ6/SN/2019 | Jin Zhou | 2019.02 | Shaanxi/China |  |  | 816 | ATG | Healthy | MW181923 |
| 24 | JZ7/SN/2019 | Jin Zhou | 2019.02 | Shaanxi/China |  |  | 822 | ATG | Healthy | MW181924 |
| 25 | TF1/SN/2016 | Teng Fei | 2016.11 | Shaanxi/China | 2037 | 954 | 822 | ATA | Healthy | MW181925 |
| 26 | TF2/SN/2016 | Teng Fei | 2016.11 | Shaanxi/China | 2032 | 948 | 813 | ATG | Healthy | MW181926 |
| 27 | TF3/SN/2016 | Teng Fei | 2016.11 | Shaanxi/China | 2032 | 948 | 813 | ATG | Healthy | MW181927 |
| 28 | TF4/SN/2016 | Teng Fei | 2016.11 | Shaanxi/China | 2035 | 948 | 816 | ATG | Healthy | MW181928 |
| 29 | TY1/SN/2016 | Tian Ying | 2016.12 | Shaanxi/China | 2037 | 954 | 822 | ATA | Healthy | MW181929 |
| 30 | TY2/SN/2016 | Tian Ying | 2016.12 | Shaanxi/China | 2037 | 954 | 822 | ATG | Healthy | MW181930 |
| 31 | TY3/SN/2016 | Tian Ying | 2016.12 | Shaanxi/China | 2044 | 948 | 828 | ATT | Healthy | MW181931 |
| 32 | SX1/SN/2017 | Sheng Xiang | 2017.05 | Shaanxi/China | 2034 | 948 | 813 | ATG | Healthy | MW181932 |
| 33 | YT1/SN/2017 | Ya Tai | 2017.06 | Shaanxi/China | 2042 | 948 | 822 | ATG | Healthy | MW181933 |
| 34 | YT2/SN/2017 | Ya Tai | 2017.06 | Shaanxi/China | 2037 | 954 | 822 | ATG | Healthy | MW181934 |
| 35 | YT3/SN/2017 | Ya Tai | 2017.06 | Shaanxi/China | 2037 | 954 | 822 | ATG | Healthy | MW181935 |
| 36 | YT4/SN/2017 | Ya Tai | 2017.06 | Shaanxi/China | 2042 | 948 | 822 | ATG | Healthy | MW181936 |
| 37 | YT5/SN/2017 | Ya Tai | 2017.06 | Shaanxi/China | 2042 | 948 | 822 | ATG | Healthy | MW181937 |
| 38 | LT1/SN/2018 | Long Teng | 2018.03 | Shaanxi/China | 2040 | 948 | 822 | ATG | Healthy | MW181938 |
| 39 | LT2/SN/2018 | Long Teng | 2018.03 | Shaanxi/China | 2039 | 954 | 825 | ATG | Healthy | MW181939 |
| 40 | LT3/SN/2018 | Long Teng | 2018.03 | Shaanxi/China | 2039 | 954 | 825 | ATG | Healthy | MW181940 |
| 41 | LT4/SN/2018 | Long Teng | 2018.03 | Shaanxi/China | 2042 | 948 | 822 | ATG | Healthy | MW181941 |
| 42 | LT5/SN/2018 | Long Teng | 2018.03 | Shaanxi/China | 2042 | 948 | 822 | ATG | Healthy | MW181942 |
| 43 | WQ1/SN/2018 | Wei Qi | 2018.03 | Shaanxi/China | 2042 | 948 | 822 | ATG | Healthy | MW181943 |
| 44 | WQ2/SN/2018 | Wei Qi | 2018.03 | Shaanxi/China | 2042 | 948 | 822 | ATG | Healthy | MW181944 |
| 45 | WQ3/SN/2018 | Wei Qi | 2018.03 | Shaanxi/China | 2037 | 954 | 822 | GTG | Healthy | MW181945 |
| 46 | WQ4/SN/2018 | Wei Qi | 2018.03 | Shaanxi/China | 2037 | 954 | 822 | ATG | Healthy | MW181946 |
| 47 | WQ5/SN/2018 | Wei Qi | 2018.03 | Shaanxi/China | 2037 | 954 | 822 | ATG | Healthy | MW181947 |
| 48 | WQ6/SN/2018 | Wei Qi | 2018.03 | Shaanxi/China | 2039 | 954 | 825 | ATG | Healthy | MW181948 |
| 49 | KW1/SN/2018 | Kai Wei | 2018.04 | Shaanxi/China | 2032 | 948 | 813 | ATG | Healthy | MW181949 |
| 50 | KW2/SN/2018 | Kai Wei | 2018.04 | Shaanxi/China | 2032 | 948 | 813 | ATG | Healthy | MW181950 |
| 51 | KW3/SN/2018 | Kai Wei | 2018.04 | Shaanxi/China | 2032 | 948 | 813 | ATG | Healthy | MW181951 |
| 52 | CA1/SN/2018 | Chang An | 2018.04 | Shaanxi/China | 2035 | 948 | 816 | ATG | Healthy | MW181952 |
| 53 | CA2/SN/2018 | Chang An | 2018.04 | Shaanxi/China | 2041 | 948 | 822 | ATG | Healthy | MW181953 |
| 54 | CA3/SN/2018 | Chang An | 2018.04 | Shaanxi/China | 2037 | 954 | 822 | ATG | Healthy | MW181954 |
| 55 | CA4/SN/2018 | Chang An | 2018.04 | Shaanxi/China | 2035 | 948 | 816 | ATG | Healthy | MW181955 |
| 56 | WL1/SN/2018 | Wei Li | 2018.05 | Shaanxi/China | 2042 | 948 | 822 | ATG | Healthy | MW181956 |
| 57 | WL2/SN/2018 | Wei Li | 2018.05 | Shaanxi/China | 2042 | 948 | 822 | ATG | Healthy | MW181957 |
| 58 | WL3/SN/2018 | Wei Li | 2018.05 | Shaanxi/China | 2042 | 948 | 822 | ATG | Healthy | MW181958 |
| 59 | WL4/SN/2018 | Wei Li | 2018.05 | Shaanxi/China | 2044 | 948 | 828 | ATT | Healthy | MW181959 |
| 60 | WL5/SN/2018 | Wei Li | 2018.05 | Shaanxi/China | 2037 | 954 | 822 | ATG | Healthy | MW181960 |
| 61 | WL6/SN/2018 | Wei Li | 2018.05 | Shaanxi/China | 2037 | 954 | 822 | ATG | Healthy | MW181961 |
| 62 | WL7/SN/2018 | Wei Li | 2018.05 | Shaanxi/China | 2037 | 954 | 822 | ATG | Healthy | MW181962 |
| 63 | QD1/SN/2018 | Qin Du | 2018.07 | Shaanxi/China | 2032 | 948 | 813 | ATG | Healthy | MW181963 |
| 64 | QD2/SN/2018 | Qin Du | 2018.07 | Shaanxi/China | 2032 | 948 | 813 | ATG | Healthy | MW181964 |
| 65 | QD3/SN/2018 | Qin Du | 2018.07 | Shaanxi/China | 2042 | 954 | 822 | ATG | Healthy | MW181965 |
| 66 | QD4/SN/2018 | Qin Du | 2018.07 | Shaanxi/China | 2037 | 954 | 822 | ATG | Healthy | MW181966 |
| 67 | QD5/SN/2018 | Qin Du | 2018.07 | Shaanxi/China | 2037 | 954 | 822 | ATG | Healthy | MW181967 |
| 68 | QD6/SN/2018 | Qin Du | 2018.07 | Shaanxi/China | 2035 | 948 | 816 | ATG | Healthy | MW181968 |
| 69 | BYHL1/BJ/2018 | Bo Ya Heng Li | 2018.08 | Beijing/China | 2032 | 948 | 813 | ATG | Healthy | MW181969 |
| 70 | DS1/GS/2018 | Ding Sheng | 2018.08 | Gansu/China | 2037 | 954 | 822 | ATA | Sick | MW181970 |
| 71 | DFSM1/QH/2018 | De Fu Si Man | 2018.08 | Qinghai/China | 2041 | 948 | 822 | ATG | Healthy | MW181971 |
| 72 | DFSM2/QH/2018 | De Fu Si Man | 2018.08 | Qinghai/China | 2041 | 948 | 822 | ATG | Healthy | MW181972 |
| 73 | JZ1/SN/2018 | Jin Zhou | 2018.08 | Shaanxi/China | 2037 | 954 | 822 | ATG | Sick | MW181973 |
| 74 | HP1/SN/2018 | Hao Peng | 2018.09 | Shaanxi/China | 2041 | 948 | 822 | ATG | Healthy | MW181974 |
| 75 | HP2/SN/2018 | Hao Peng | 2018.09 | Shaanxi/China | 2037 | 954 | 822 | ATG | Healthy | MW181975 |
| 76 | HP3/SN/2018 | Hao Peng | 2018.09 | Shaanxi/China | 2032 | 948 | 813 | ATG | Healthy | MW181976 |
| 77 | HP4/SN/2018 | Hao Peng | 2018.09 | Shaanxi/China | 2035 | 948 | 816 | ATG | Healthy | MW181977 |
| 78 | YB1/SN/2018 | Yi Bai | 2018.10 | Shaanxi/China | 2042 | 954 | 825 | ATG | Healthy | MW181978 |
| 79 | YB2/SN/2018 | Yi Bai | 2018.10 | Shaanxi/China | 2037 | 954 | 822 | ATG | Healthy | MW181979 |
| 80 | YB3/SN/2018 | Yi Bai | 2018.10 | Shaanxi/China | 2037 | 954 | 822 | ATG | Healthy | MW181980 |
| 81 | YB4/SN/2018 | Yi Bai | 2018.10 | Shaanxi/China | 2034 | 948 | 813 | ATG | Healthy | MW181981 |
| 82 | DA1/XJ/2018 | Da Ai | 2018.10 | Xinjiang/China | 2030 | 954 | 813 | ATG | Sick | MW181982 |
| 83 | LQ1/SN/2018 | Li Quan | 2018.12 | Shaanxi/China | 2035 | 948 | 816 | ATG | Healthy | MW181983 |
| 84 | LQ2/SN/2018 | Li Quan | 2018.12 | Shaanxi/China | 2041 | 948 | 822 | ATG | Healthy | MW181984 |
| 85 | LQ3/SN/2018 | Li Quan | 2018.12 | Shaanxi/China | 2045 | 948 | 825 | ATG | Healthy | MW181985 |
| 86 | JZ2/SN/2019 | Jin Zhou | 2019.02 | Shaanxi/China | 2035 | 948 | 816 | ATG | Healthy | MW181986 |
| 87 | JZ3/SN/2019 | Jin Zhou | 2019.02 | Shaanxi/China | 2041 | 948 | 822 | ATG | Healthy | MW181987 |
| 88 | LH2/HE/2019 | Ling Hang | 2019.04 | Hebei/China | 2037 | 954 | 822 | ATA | Sick | MW181988 |
| 89 | LH3/HE/2019 | Ling Hang | 2019.04 | Hebei/China | 2042 | 948 | 822 | ATG | Sick | MW181989 |
| 90 | QD7/SN/2019 | Qin Du | 2019.04 | Shaanxi/China | 2037 | 954 | 822 | ATA | Sick | MW181990 |
| 91 | QD8/SN/2019 | Qin Du | 2019.08 | Shaanxi/China | 2035 | 948 | 816 | ATG | Healthy | MW181991 |
