# Supplementary material for: Molecular detection and phylogenetic analysis of pigeon circovirus from racing pigeons in Northern China
Source: BMC Genomics. 2022 Apr 11;23:290. doi: 10.1186/s12864-022-08425-8 (PMC8995411; doi:10.1186/s12864-022-08425-8)
Supplement: Supplementary file 3 — Additional file 3: Table S3. Sequence analysis of genome sequences of Pigeon circovirus (PiCV) strains. The information including homology analysis of amino acid and nucleotide sequences. [file 12864_2022_8425_MOESM3_ESM.docx]

**Supplementary Table 3** Sequence analysis of genome sequences of Pigeon circovirus (PiCV) strains.

| Selected strains | Genome sequence | *cap* gene sequence | | *rep* gene sequence | |
| --- | --- | --- | --- | --- | --- |
|  | Nucleotide | Nucleotide | Amino acids | Nucleotide | Amino acids |
| Identity of the PiCV strains identified in our study | 84.2%–100% | 71.9%–100% | 71.7%–100% | 90.3%–100% | 92.7%–100% |
| Compared with Chinese PiCV reference strains | 83.0%–97.8% | 73.0%–99.6% | 72.3%–100% | 89.0%–99.2% | 89.2%–99.6% |
| Compared with PiCV reference strains from other countries | 82.0%–98.3% | 68.8%–98.4% | 63.6%–100% | 89.5%–98.3% | 90.5%–99.3% |
| ***Note.*** The GenBank accession no. of the Chinese PiCV reference strains, PiCV reference strains from other countries, and the identified PiCV strains was shown in Table S1 and S2. | | | | | |
