# Supplementary material for: Molecular detection and phylogenetic analysis of pigeon circovirus from racing pigeons in Northern China
Source: BMC Genomics. 2022 Apr 11;23:290. doi: 10.1186/s12864-022-08425-8 (PMC8995411; doi:10.1186/s12864-022-08425-8)
Supplement: Supplementary file 4 — Additional file 4: Table S4. Details of recombination events (GenBank accession numbers) detected in the Pigeon circovirus (PiCV) strains obtained in this study. The information including location of recombination breakpoints, recombinant, potential minor parent(s), potential major parent(s), detection methods and p-value. [file 12864_2022_8425_MOESM4_ESM.docx]

**Supplementary Table 4** Details of recombination events (GenBank accession numbers) detected in the Pigeon circovirus (PiCV) strains obtained in this study.

| Event | Genome position | | Recombinant | | Potential minor parent(s) | | | | Potential major parent(s) | | | | Detection methods | *p*-value |
| --- | --- | --- | --- | --- | --- | --- | --- | --- | --- | --- | --- | --- | --- | --- |
| 1 | 1518 | 2019 | MF136688 |  | KF738857 |  |  |  | MF136684 | KX108792 | KF738856 | KF738851 | GBMCS**T** | 1.05×10^-41^ |
|  |  |  |  |  | KF738844 |  |  |  | AF252610 | KX108794 | KF738859 | KF738852 |  |  |
|  |  |  |  |  | KF738850 |  |  |  | **MW181970** | KX108795 | KF738862 | KF738853 |  |  |
|  |  |  |  |  | MF136690 |  |  |  | **MW181925** | KX108801 | KF738863 | DQ915962 |  |  |
|  |  |  |  |  |  |  |  |  | **MW181929** | KX108808 | KF738864 | KX108782 |  |  |
|  |  |  |  |  |  |  |  |  | **MW181965** | KX108814 | KF738866 | MF136680 |  |  |
|  |  |  |  |  |  |  |  |  | NC002361 | KX108815 | KF738868 | MF136682 |  |  |
|  |  |  |  |  |  |  |  |  | AJ298230 | KX108820 | KF738870 | MF136686 |  |  |
|  |  |  |  |  |  |  |  |  | AJ298229 | KX108822 | KF738871 | MF136687 |  |  |
|  |  |  |  |  |  |  |  |  | DQ915960 | KF738869 | KF738872 | MF136691 |  |  |
|  |  |  |  |  |  |  |  |  | KX108789 | KF738846 | KF738845 | MF136692 |  |  |
| 2 | 995 | 1996 | **MW181940** |  | **MW181941** |  |  |  | KX108804 | **MW181990** | KX108794 | KX108815 | RGBMS**T** | 1.25×10^-35^ |
|  |  |  | **MW181939** |  | **MW181984** |  |  |  | AF252610 | NC002361 | KX108801 | KX108820 |  |  |
|  |  |  | **MW181948** |  | **MW181953** |  |  |  | **MW181970** | AJ298229 | KX108808 | KX108822 |  |  |
|  |  |  | KX108807 |  | **MW181956** |  |  |  | **MW181988** | DQ915960 | KX108811 | KF738868 |  |  |
|  |  |  | KX108812 |  | **MW181987** |  |  |  | **MW181935** | KX108786 | KX108813 |  |  |  |
| 3 | 134 | 1482 | MF136690 |  | KX108822 | KX108786 | KX108814 | KF738866 | KF738850 |  |  |  | GBMCS**T** | 1.59×10^-30^ |
|  |  |  |  |  | AF252610 | KX108789 | KX108819 | KF738868 | DQ915950 |  |  |  |  |  |
|  |  |  |  |  | **MW181970** | KX108792 | KX108820 | KF738870 | KF738857 |  |  |  |  |  |
|  |  |  |  |  | **MW181988** | KX108794 | KX108823 | KF738871 | KF738844 |  |  |  |  |  |
|  |  |  |  |  | **MW181925** | KX108795 | KX108824 | KF738872 |  |  |  |  |  |  |
|  |  |  |  |  | **MW181929** | KX108799 | KF738869 | KF738848 |  |  |  |  |  |  |
|  |  |  |  |  | **MW181965** | KX108801 | KF738856 | KF738853 |  |  |  |  |  |  |
|  |  |  |  |  | **MW181945** | KX108802 | KF738859 | DQ915961 |  |  |  |  |  |  |
|  |  |  |  |  | **MW181990** | KX108803 | KF738861 | DQ915962 |  |  |  |  |  |  |
|  |  |  |  |  | NC002361 | KX108804 | KF738863 | KX108782 |  |  |  |  |  |  |
|  |  |  |  |  | AJ298229 | KX108808 | KF738864 | MF136692 |  |  |  |  |  |  |
|  |  |  |  |  | DQ915960 | KX108811 | KF738865 |  |  |  |  |  |  |  |
| 4 | 1136 | 1989 | ^‡^KX108827 |  | **MW181944** | **MW181941** | **MW181956** |  | Unknown |  |  |  | RGBMCS**T** | 3.16×10^-30^ |
|  |  |  |  |  | **MW181942** | **MW181957** | **MW181958** |  |  |  |  |  |  |  |
| 5 | 1349 | 2018 | JN183455 |  | DQ915950 |  |  |  | KX108801 | KX108786 | KX108815 | KF738865 | GBMCS**T** | 2.34×10^-28^ |
|  |  |  |  |  | MF621931 |  |  |  | AF252610 | KX108787 | KX108819 | KF738866 |  |  |
|  |  |  |  |  | MF664483 |  |  |  | **MW181970** | KX108789 | KX108820 | KF738868 |  |  |
|  |  |  |  |  | DQ915956 |  |  |  | **MW181988** | KX108792 | KX108822 | KF738871 |  |  |
|  |  |  |  |  | KF738857 |  |  |  | **MW181925** | KX108794 | KX108824 | KF738872 |  |  |
|  |  |  |  |  | KF738844 |  |  |  | **MW181929** | KX108795 | KF738869 | KF738845 |  |  |
|  |  |  |  |  | KF738850 |  |  |  | **MW181945** | KX108799 | KF738846 | KF738848 |  |  |
|  |  |  |  |  |  |  |  |  | **MW181990** | KX108802 | KF738856 | KF738851 |  |  |
|  |  |  |  |  |  |  |  |  | NC002361 | KX108803 | KF738859 | KF738852 |  |  |
|  |  |  |  |  |  |  |  |  | AJ298230 | KX108804 | KF738861 | KF738853 |  |  |
|  |  |  |  |  |  |  |  |  | AJ298229 | KX108808 | KF738862 | DQ915961 |  |  |
|  |  |  |  |  |  |  |  |  | DQ915960 | KX108811 | KF738863 | DQ915962 |  |  |
|  |  |  |  |  |  |  |  |  | KX108784 | KX108814 | KF738864 | KX108782 |  |  |
| 6 | 1006 | 2016 | ^‡^KF738865 |  | KF738845 | NC002361 | KX108808 | KF738872 | Unknown |  |  |  | RGBM**S**T | 1.47×10^-30^ |
|  |  |  | KF738869 |  | AF252610 | AJ298230 | KX108819 | KF738848 |  |  |  |  |  |  |
|  |  |  | KF738846 |  | **MW181970** | AJ298229 | KX108822 | KF738851 |  |  |  |  |  |  |
|  |  |  | KF738856 |  | **MW181988** | DQ915960 | KX108824 | KF738852 |  |  |  |  |  |  |
|  |  |  | KF738862 |  | **MW181925** | KX108786 | KF738859 | KF738853 |  |  |  |  |  |  |
|  |  |  | KF738863 |  | **MW181929** | KX108789 | KF738861 | DQ915962 |  |  |  |  |  |  |
|  |  |  | KF738864 |  | **MW181965** | KX108795 | KF738868 |  |  |  |  |  |  |  |
|  |  |  |  |  | **MW181990** | KX108803 | KF738871 |  |  |  |  |  |  |  |
| 7 | 763 | 2016 | **MW181935** |  | **MW181973** | **MW181967** | **MW181960** | **MW181946** | KX108804 | KX108817 |  |  | GBMC**S**T | 1.51×10^-28^ |
|  |  |  | **MW181930** |  | **MW181954** | **MW181966** | **MW181934** | **MW181980** | KX108800 | KX108823 |  |  |  |  |
|  |  |  |  |  | **MW181975** | **MW181962** | **MW181947** | **MW181979** | KX108811 |  |  |  |  |  |
| 8 | 1137 | 2006 | KX108806 | KX108805 | **MW181968** | **MW181928** |  |  | KX108824 | KX108787 | KX108810 | KF738872 | RGMCS**T** | 7.99×10^-25^ |
|  |  |  | **MW181991** | KX108826 | **MW181949** | **MW181977** |  |  | **MW181929** | KX108801 | KX108814 | KF738845 |  |  |
|  |  |  |  |  | **MW181950** | KX431143 |  |  | DQ915960 | KX108802 | KX108819 | KF738853 |  |  |
|  |  |  |  |  | **MW181951** |  |  |  | KX108783 | KX108803 | KX108822 | DQ915961 |  |  |
|  |  |  |  |  | **MW181952** |  |  |  | KX108785 | KX108804 | KF738861 |  |  |  |
| 9 | 134 | 1264 | ^‡^DQ915950 |  | Unknown |  |  |  | DQ915956 | KY114965 | **MW181931** | KF738857 | RGBMCS**T** | 9.43×10^-23^ |
|  |  |  |  |  |  |  |  |  | KX808543 | MF664483 | **MW181959** | KF738844 |  |  |
| 10 | 2020 | 1155 | DQ090944 |  | KX108801 | KX108780 | KX108803 | KF738859 | **MW181941** |  |  |  | RMC**T** | 2.32×10^-22^ |
|  |  |  |  |  | AF252610 | KX108783 | KX108804 | KF738861 | **MW181938** |  |  |  |  |  |
|  |  |  |  |  | MG518478 | KX108784 | KX108808 | KF738866 | **MW181942** |  |  |  |  |  |
|  |  |  |  |  | **MW181970** | KX108785 | KX108810 | KF738867 | **MW181984** |  |  |  |  |  |
|  |  |  |  |  | **MW181988** | KX108786 | KX108811 | KF738868 | **MW181953** |  |  |  |  |  |
|  |  |  |  |  | **MW181925** | KX108787 | KX108813 | KF738870 | **MW181974** |  |  |  |  |  |
|  |  |  |  |  | **MW181929** | KX108788 | KX108814 | KF738871 | **MW181957** |  |  |  |  |  |
|  |  |  |  |  | **MW181934** | KX108792 | KX108815 | KF738872 | **MW181956** |  |  |  |  |  |
|  |  |  |  |  | **MW181945** | KX108793 | KX108816 | KF738848 | **MW181958** |  |  |  |  |  |
|  |  |  |  |  | **MW181980** | KX108794 | KX108818 | KF738851 | **MW181933** |  |  |  |  |  |
|  |  |  |  |  | **MW181979** | KX108795 | KX108819 | KF738852 | **MW181936** |  |  |  |  |  |
|  |  |  |  |  | **MW181990** | KX108797 | KX108820 | DQ915961 | **MW181937** |  |  |  |  |  |
|  |  |  |  |  | NC002361 | KX108798 | KX108821 | DQ915962 | **MW181944** |  |  |  |  |  |
|  |  |  |  |  | AJ298230 | KX108799 | KX108822 | KX108782 | **MW181943** |  |  |  |  |  |
|  |  |  |  |  | AJ298229 | KX108800 | KX108824 |  | **MW181987** |  |  |  |  |  |
|  |  |  |  |  | DQ915960 | KX108802 | KF738847 |  | **MW181989** |  |  |  |  |  |
| 11 | 98 | 1312 | ^‡^KF738850 |  | KX108819 | DQ915960 | KX108808 | KF738866 | KF738844 |  |  |  | RBMC**T** | 4.21×10^-21^ |
|  |  |  |  |  | AF252610 | KX108786 | KX108811 | KF738868 | KF738857 |  |  |  |  |  |
|  |  |  |  |  | **MW181970** | KX108789 | KX108814 | KF738870 |  |  |  |  |  |  |
|  |  |  |  |  | **MW181988** | KX108794 | KX108815 | KF738872 |  |  |  |  |  |  |
|  |  |  |  |  | **MW181925** | KX108795 | KX108817 | KF738845 |  |  |  |  |  |  |
|  |  |  |  |  | **MW181929** | KX108799 | KX108820 | KF738848 |  |  |  |  |  |  |
|  |  |  |  |  | **MW181965** | KX108800 | KX108822 | KF738851 |  |  |  |  |  |  |
|  |  |  |  |  | **MW181945** | KX108801 | KX108823 | KF738852 |  |  |  |  |  |  |
|  |  |  |  |  | **MW181990** | KX108802 | KX108824 | KF738853 |  |  |  |  |  |  |
|  |  |  |  |  | NC002361 | KX108803 | KF738859 | DQ915961 |  |  |  |  |  |  |
|  |  |  |  |  | AJ298229 | KX108804 | KF738861 | KX108782 |  |  |  |  |  |  |
| 12 | 1181 | 1915 | **^‡^MW181972** |  | LC035390 |  |  |  | **MW181989** | **MW181953** | **MW181936** | KF738849 | RGBM**S**T | 1.38×10^-24^ |
|  |  |  | **MW181971** |  |  |  |  |  | MF664482 | **MW181974** | **MW181937** | DQ090945 |  |  |
|  |  |  |  |  |  |  |  |  | **MW181938** | **MW181957** | **MW181944** | DQ090944 |  |  |
|  |  |  |  |  |  |  |  |  | **MW181942** | **MW181956** | **MW181943** |  |  |  |
|  |  |  |  |  |  |  |  |  | **MW181941** | **MW181958** | **MW181987** |  |  |  |
|  |  |  |  |  |  |  |  |  | **MW181984** | **MW181933** | KF738858 |  |  |  |
| 13 | 255 | 2030 | **^‡^MW181985** | **MW181944** | KF738854 |  |  |  | LC035390 |  |  |  | RGBMC**S**T | 1.39×10^-34^ |
|  |  |  | **MW181969** | **MW181981** |  |  |  |  |  |  |  |  |  |  |
|  |  |  | **MW181955** | **MW181932** |  |  |  |  |  |  |  |  |  |  |
|  |  |  | **MW181926** | **MW181989** |  |  |  |  |  |  |  |  |  |  |
|  |  |  | **MW181976** | DQ915957 |  |  |  |  |  |  |  |  |  |  |
|  |  |  | **MW181974** |  |  |  |  |  |  |  |  |  |  |  |
| 14 | 80 | 981 | ^‡^MF136688 |  | MF136689 |  |  |  | KF738848 | KX108780 | KX108801 | KF738859 | RGBMCS**T** | 2.70×10^-19^ |
|  |  |  | MF136680 |  | DQ915959 |  |  |  | AF252610 | KX108783 | KX108803 | KF738861 |  |  |
|  |  |  | MF136682 |  |  |  |  |  | MG518478 | KX108784 | KX108804 | KF738867 |  |  |
|  |  |  | MF136684 |  |  |  |  |  | **MW181970** | KX108785 | KX108808 | KF738868 |  |  |
|  |  |  | MF136686 |  |  |  |  |  | **MW181988** | KX108786 | KX108811 | KF738871 |  |  |
|  |  |  | MF136687 |  |  |  |  |  | **MW181925** | KX108787 | KX108815 | KF738872 |  |  |
|  |  |  | MF136691 |  |  |  |  |  | **MW181945** | KX108788 | KX108816 | KF738845 |  |  |
|  |  |  |  |  |  |  |  |  | **MW181990** | KX108789 | KX108819 | KF738851 |  |  |
|  |  |  |  |  |  |  |  |  | NC002361 | KX108793 | KX108820 | KF738852 |  |  |
|  |  |  |  |  |  |  |  |  | AJ298230 | KX108794 | KX108822 | KF738853 |  |  |
|  |  |  |  |  |  |  |  |  | AJ298229 | KX108795 | KX108824 | DQ915961 |  |  |
|  |  |  |  |  |  |  |  |  | DQ915960 | KX108800 | KF738847 | DQ915962 |  |  |
| 15 | 2029 | 124 | **MW181931** | DQ915956 | LC035390 |  |  |  | KX108783 | KX108784 | KX108803 | KF738859 | **R**GBMCST | 2.85×10^-17^ |
|  |  |  | KX808543 | DQ915950 |  |  |  |  | AF252610 | KX108785 | KX108804 | KF738861 |  |  |
|  |  |  | KY114965 | KF738857 |  |  |  |  | MG518478 | KX108786 | KX108808 | KF738866 |  |  |
|  |  |  | MF621931 | KF738860 |  |  |  |  | **MW181970** | KX108787 | KX108810 | KF738867 |  |  |
|  |  |  | MF664483 | KF738844 |  |  |  |  | **MW181988** | KX108788 | KX108811 | KF738868 |  |  |
|  |  |  | **MW181959** | MF136690 |  |  |  |  | **MW181925** | KX108789 | KX108813 | KF738870 |  |  |
|  |  |  |  |  |  |  |  |  | **MW181929** | KX108790 | KX108814 | KF738871 |  |  |
|  |  |  |  |  |  |  |  |  | **MW181965** | KX108792 | KX108815 | KF738872 |  |  |
|  |  |  |  |  |  |  |  |  | **MW181945** | KX108793 | KX108816 | KF738845 |  |  |
|  |  |  |  |  |  |  |  |  | **MW181990** | KX108794 | KX108818 | KF738848 |  |  |
|  |  |  |  |  |  |  |  |  | NC002361 | KX108795 | KX108819 | KF738851 |  |  |
|  |  |  |  |  |  |  |  |  | AJ298230 | KX108798 | KX108820 | KF738852 |  |  |
|  |  |  |  |  |  |  |  |  | AJ298229 | KX108799 | KX108821 | KF738853 |  |  |
|  |  |  |  |  |  |  |  |  | DQ915960 | KX108800 | KX108822 | DQ915961 |  |  |
|  |  |  |  |  |  |  |  |  | KX108780 | KX108801 | KX108824 | DQ915962 |  |  |
|  |  |  |  |  |  |  |  |  | KX108781 | KX108802 | KF738847 | KX108782 |  |  |
| 16 | 1259 | 1968 | KF738867 | KF738857 | Unknown |  |  |  | KF738871 | KX108795 | KF738859 | KF738853 | M**S**T | 5.09×10^-21^ |
|  |  |  | DQ915956 | KF738844 |  |  |  |  | AF252610 | KX108801 | KF738861 | MF136680 |  |  |
|  |  |  | KF738847 |  |  |  |  |  | **MW181970** | KX108802 | KF738866 | MF136682 |  |  |
|  |  |  |  |  |  |  |  |  | **MW181988** | KX108803 | KF738868 | MF136684 |  |  |
|  |  |  |  |  |  |  |  |  | **MW181990** | KX108815 | KF738870 | MF136686 |  |  |
|  |  |  |  |  |  |  |  |  | NC002361 | KX108819 | KF738872 | MF136687 |  |  |
|  |  |  |  |  |  |  |  |  | AJ298229 | KX108820 | KF738845 | MF136691 |  |  |
|  |  |  |  |  |  |  |  |  | DQ915960 | KX108822 | KF738848 |  |  |  |
|  |  |  |  |  |  |  |  |  | KX108786 | KX108824 | KF738852 |  |  |  |
| 17 | 1005 | 2008 | ^‡^KX108809 |  | Unknown |  |  |  | **MW181929** |  |  |  | RGMC**S**T | 8.03×10^-18^ |
|  |  |  |  |  |  |  |  |  |  |  |  |  |  |  |
| 18 | 102 | 1005^†^ | **^‡^MW181991** |  | KX108805 |  |  |  | KF738865 | KF738856 | KF738863 |  | MS**T** | 1.60×10^-12^ |
|  |  |  |  |  |  |  |  |  | KF738846 | KF738862 | KF738864 |  |  |  |
| 19 | 1155 | 1901 | ^‡^MW181965 |  | KX108801 | DQ915960 | KX108822 | KF738861 | Unknown |  |  |  | M**S**T | 1.11×10^-13^ |
|  |  |  |  |  | **MW181970** | KX108795 | KX108824 | KF738871 |  |  |  |  |  |  |
|  |  |  |  |  | **MW181925** | KX108819 | KF738859 | KF738848 |  |  |  |  |  |  |
| 20^§^ | 459 | 573 | ^‡^KX108827 |  | Unknown |  |  |  | **MW181945** | KX108795 | KX108814 | KF738859 | **G**MCST | 1.55×10^-9^ |
|  |  |  |  |  |  |  |  |  | AF252610 | KX108796 | KX108815 | KF738867 |  |  |
|  |  |  |  |  |  |  |  |  | **MW181970** | KX108801 | KX108818 | KF738868 |  |  |
|  |  |  |  |  |  |  |  |  | **MW181925** | KX108802 | KX108819 | KF738843 |  |  |
|  |  |  |  |  |  |  |  |  | **MW181929** | KX108803 | KX108820 | KF738845 |  |  |
|  |  |  |  |  |  |  |  |  | NC002361 | KX108805 | KX108822 | KF738851 |  |  |
|  |  |  |  |  |  |  |  |  | AJ298229 | KX108806 | KX108824 | KF738852 |  |  |
|  |  |  |  |  |  |  |  |  | DQ915956 | KX108809 | KX108825 | KF738853 |  |  |
|  |  |  |  |  |  |  |  |  | KX108792 | KX108810 | KX108826 | KX108782 |  |  |
|  |  |  |  |  |  |  |  |  | KX108794 | KX108811 | KF738847 |  |  |  |
|  |  |  |  |  |  |  |  |  |  |  |  |  |  |  |
| 21^§^ | 272 | 1131 | ^‡^MF136681 |  | KX108804 | AJ298229 | KX108803 | KF738867 | DQ915959 |  |  |  | RGMC**T** | 1.99×10^-8^ |
|  |  |  |  |  | KY114965 | DQ915960 | KX108805 | KF738868 | MF136689 |  |  |  |  |  |
|  |  |  |  |  | MF621931 | KX108780 | KX108808 | KF738870 |  |  |  |  |  |  |
|  |  |  |  |  | MF664483 | KX108783 | KX108810 | KF738871 |  |  |  |  |  |  |
|  |  |  |  |  | MG518478 | KX108784 | KX108811 | KF738872 |  |  |  |  |  |  |
|  |  |  |  |  | **MW181970** | KX108786 | KX108813 | KF738844 |  |  |  |  |  |  |
|  |  |  |  |  | **MW181925** | KX108787 | KX108814 | KF738845 |  |  |  |  |  |  |
|  |  |  |  |  | **MW181975** | KX108788 | KX108816 | KF738848 |  |  |  |  |  |  |
|  |  |  |  |  | **MW181959** | KX108789 | KX108817 | KF738852 |  |  |  |  |  |  |
|  |  |  |  |  | **MW181962** | KX108792 | KX108819 | KF738853 |  |  |  |  |  |  |
|  |  |  |  |  | **MW181960** | KX108793 | KX108820 | DQ915961 |  |  |  |  |  |  |
|  |  |  |  |  | **MW181961** | KX108794 | KX108822 | DQ915962 |  |  |  |  |  |  |
|  |  |  |  |  | **MW181980** | KX108795 | KX108823 | DQ090945 |  |  |  |  |  |  |
|  |  |  |  |  | **MW181973** | KX108799 | KX108824 | KX108782 |  |  |  |  |  |  |
|  |  |  |  |  | **MW181990** | KX108800 | KF738857 | JN183455 |  |  |  |  |  |  |
|  |  |  |  |  | **MW181982** | KX108801 | KF738859 |  |  |  |  |  |  |  |
|  |  |  |  |  | NC002361 | KX108802 | KF738861 |  |  |  |  |  |  |  |
|  |  |  |  |  |  |  |  |  |  |  |  |  |  |  |
|  |  |  |  |  |  |  |  |  |  |  |  |  |  |  |
| 22 | 86 | 1004^†^ | KF738869 |  | KF738846 |  |  |  | MG518478 | AJ298230 | KX108817 | KF738851 | MS**T** | 5.08×10^-8^ |
|  |  |  |  |  | KF738865 |  |  |  | AF252610 | DQ915960 | KX108820 | KF738852 |  |  |
|  |  |  |  |  |  |  |  |  | **MW181970** | KX108786 | KX108825 | KF738853 |  |  |
|  |  |  |  |  |  |  |  |  | **MW181988** | KX108789 | KF738847 | DQ915961 |  |  |
|  |  |  |  |  |  |  |  |  | **MW181925** | KX108795 | KF738859 | DQ915962 |  |  |
|  |  |  |  |  |  |  |  |  | **MW181935** | KX108800 | KF738868 | JN183455 |  |  |
|  |  |  |  |  |  |  |  |  | **MW181990** | KX108807 | KF738871 |  |  |  |
|  |  |  |  |  |  |  |  |  | **MW181982** | KX108808 | KF738845 |  |  |  |
|  |  |  |  |  |  |  |  |  | NC002361 | KX108815 | KF738848 |  |  |  |
| 23 | 158^†^ | 1227 | ^‡^KF738861 |  | KY114965 | KX808543 | MF621931 | MF664483 | KF738871 | AJ298229 |  |  | **M**CT | 9.03×10^-8^ |
| 24 | 113 | 467 | ^‡^LC035390 |  | **MW181978** |  |  |  | Unknown |  |  |  | MC**T** | 1.50×10^-7^ |
| 25 | 176 | 1152 | **^‡^MW181960** | **MW181934** | KX108789 |  |  |  | Unknown |  |  |  | MCS**T** | 1.30×10^-6^ |
|  |  |  | **MW181954** | **MW181947** | **MW181988** |  |  |  |  |  |  |  |  |  |
|  |  |  | **MW181975** | **MW181946** | **MW181990** |  |  |  |  |  |  |  |  |  |
|  |  |  | **MW181967** | **MW181980** | KX108824 |  |  |  |  |  |  |  |  |  |
|  |  |  | **MW181966** | **MW181979** | KF738859 |  |  |  |  |  |  |  |  |  |
|  |  |  | **MW181962** | **MW181973** | JN183455 |  |  |  |  |  |  |  |  |  |
|  |  |  | **MW181961** | KX108784 |  |  |  |  |  |  |  |  |  |  |
| 26 | 1457 | 1777 | KX108799 |  | DQ915950 |  |  |  | KF738853 | KX108789 | KF738868 | MF136680 | M**C**ST | 1.25×10^-6^ |
|  |  |  |  |  | KF738850 |  |  |  | AF252610 | KX108792 | KF738870 | MF136682 |  |  |
|  |  |  |  |  |  |  |  |  | **MW181970** | KX108801 | KF738871 | MF136684 |  |  |
|  |  |  |  |  |  |  |  |  | **MW181925** | KX108814 | KF738872 | MF136686 |  |  |
|  |  |  |  |  |  |  |  |  | NC002361 | KX108822 | KF738851 | MF136691 |  |  |
|  |  |  |  |  |  |  |  |  | AJ298230 | KF738859 | KF738852 | MF136692 |  |  |
|  |  |  |  |  |  |  |  |  | AJ298229 | KF738861 | DQ915962 |  |  |  |
|  |  |  |  |  |  |  |  |  | DQ915960 | KF738866 | KX108782 |  |  |  |
| 27 | 86 | 253^†^ | **MW181929** | KX108798 | **MW181974** |  |  |  | KX108789 | DQ915960 | KX108807 | KF738859 | MC**S**T | 2.10×10^-6^ |
|  |  |  | KX108781 | KX108818 | **MW181986** |  |  |  | AF252610 | KX108780 | KX108808 | KF738867 |  |  |
|  |  |  | KX108790 | KX108821 |  |  |  |  | MG518478 | KX108784 | KX108810 | KF738868 |  |  |
|  |  |  | KX108796 |  |  |  |  |  | **MW181970** | KX108786 | KX108811 | KF738871 |  |  |
|  |  |  |  |  |  |  |  |  | **MW181988** | KX108787 | KX108812 | KF738845 |  |  |
|  |  |  |  |  |  |  |  |  | **MW181939** | KX108788 | KX108814 | KF738848 |  |  |
|  |  |  |  |  |  |  |  |  | **MW181940** | KX108792 | KX108815 | KF738851 |  |  |
|  |  |  |  |  |  |  |  |  | **MW181925** | KX108794 | KX108817 | KF738852 |  |  |
|  |  |  |  |  |  |  |  |  | **MW181948** | KX108795 | KX108820 | KF738853 |  |  |
|  |  |  |  |  |  |  |  |  | **MW181990** | KX108799 | KX108822 | DQ915961 |  |  |
|  |  |  |  |  |  |  |  |  | **MW181982** | KX108800 | KX108823 | DQ915962 |  |  |
|  |  |  |  |  |  |  |  |  | NC002361 | KX108801 | KX108824 | KX108782 |  |  |
|  |  |  |  |  |  |  |  |  | AJ298230 | KX108804 | KX108825 | JN183455 |  |  |
|  |  |  |  |  |  |  |  |  | AJ298229 | KX108806 | KF738847 |  |  |  |
| 28 | 79 | 253^†^ | KX108809 | KX108816 | KX431143 |  |  |  | KF738853 | AJ298229 | KX108810 | KF738868 | MS**T** | 1.34×10^-5^ |
|  |  |  | KX108793 | KX108826 | **MW181949** |  |  |  | AF252610 | DQ915960 | KX108811 | KF738872 |  |  |
|  |  |  | KX108797 | KF738854 | **MW181950** |  |  |  | MG518478 | KX108786 | KX108815 | KF738843 |  |  |
|  |  |  |  |  | **MW181951** |  |  |  | **MW181970** | KX108789 | KX108817 | KF738845 |  |  |
|  |  |  |  |  | **MW181952** |  |  |  | **MW181988** | KX108795 | KX108820 | KF738848 |  |  |
|  |  |  |  |  | **MW181927** |  |  |  | **MW181925** | KX108799 | KX108822 | KF738851 |  |  |
|  |  |  |  |  | **MW181977** |  |  |  | **MW181990** | KX108800 | KX108824 | KF738852 |  |  |
|  |  |  |  |  | **MW181976** |  |  |  | **MW181982** | KX108801 | KF738847 | DQ915961 |  |  |
|  |  |  |  |  | **MW181974** |  |  |  | NC002361 | KX108807 | KF738859 | DQ915962 |  |  |
|  |  |  |  |  | **MW181981** |  |  |  | AJ298230 | KX108808 | KF738867 | JN183455 |  |  |
|  |  |  |  |  | **MW181932** |  |  |  |  |  |  |  |  |  |
|  |  |  |  |  | **MW181986** |  |  |  |  |  |  |  |  |  |
|  |  |  |  |  | DQ915957 |  |  |  |  |  |  |  |  |  |
| 29 | 222 | 711^†^ | **^‡^MW181970** |  | KX108791 |  |  |  | DQ915962 | AJ298230 |  |  | M**S**T | 3.13×10^-4^ |
| 30^§^ | 398 | 895^†^ | ^‡^MF136689 |  | **MW181931** | KX108780 | KX108789 | KX108822 | Unknown |  |  |  | MC**T** | 1.48×10^-2^ |
|  |  |  |  |  | **MW181988** | KX108785 | KX108799 | DQ915961 |  |  |  |  |  |  |
|  |  |  |  |  | **MW181959** | KX108786 | KX108801 | DQ915962 |  |  |  |  |  |  |
|  |  |  |  |  | **MW181990** | KX108787 | KX108804 |  |  |  |  |  |  |  |
|  |  |  |  |  | AJ298230 | KX108788 | KX108816 |  |  |  |  |  |  |  |
| 31^§^ | 1473 | 85^†^ | ^‡^KX108811 |  | Unknown |  |  |  | **MW181929** |  |  |  | M**S**T | 2.59×10^-9^ |

***Note.*** Methods used to detect recombination are RDP (R), GENCONV (G), Bootscan (B), MaxChi (M), Chimaera (C), Siscan (S) and 3Seq (T). The method with the most significant associated *p*-value is indicated in bold for each event. The GenBank accession numbers related to PiCV isolates in this study are highlighted in bold font.

^†^The actual breakpoint position is undetermined.

^‡^The recombinant sequence may have been misidentified.

^§^The recombination signal could have been caused by an evolutionary process other than recombination.
